# Combinatorial characterizations and impossibilities for higher-order homophily

**DOI:** 10.1126/sciadv.abq3200

**Published:** 2023-01-06

**Authors:** Nate Veldt, Austin R. Benson, Jon Kleinberg

**Affiliations:** ^1^ Department of Computer Science and Engineering, Texas A&M University, College Station, TX 77843, USA.; ^2^ Department of Computer Science, Cornell University, Ithaca, NY 14853, USA.

## Abstract

Homophily is the seemingly ubiquitous tendency for people to connect and interact with other individuals who are similar to them. This is a well-documented principle and is fundamental for how society organizes. Although many social interactions occur in groups, homophily has traditionally been measured using a graph model, which only accounts for pairwise interactions involving two individuals. Here, we develop a framework using hypergraphs to quantify homophily from group interactions. This reveals natural patterns of group homophily that appear with gender in scientific collaboration and political affiliation in legislative bill cosponsorship and also reveals distinctive gender distributions in group photographs, all of which cannot be fully captured by pairwise measures. At the same time, we show that seemingly natural ways to define group homophily are combinatorially impossible. This reveals important pitfalls to avoid when defining and interpreting notions of group homophily, as higher-order homophily patterns are governed by combinatorial constraints that are independent of human behavior but are easily overlooked.

## Teaser

In group interactions, the tendency for “birds of a feather to flock together” is governed by surprising mathematical limits.

## INTRODUCTION

Homophily is the established sociological principle that individuals tend to associate and form connections with other individuals that are similar to them (*1*). For example, social ties are strongly correlated with demographic factors such as race, age, and gender (*2*–*4*); acquired characteristics such as education, political affiliation, and religion (*4*, *5*); and even psychological factors such as attitudes and aspirations (*6*, *7*). For many of these factors, homophily persists across a wide range of relationship types, from marriage (*8*), to friendships (*9*), to ties based simply on whether individuals have been observed together in public (*10*). As a consequence, homophily serves as an important concept for understanding human relationships and social connections and is a key guiding principle for research in sociology and network analysis.

A major motivation in homophily research is to understand how similarity among individuals influences group formation and group interactions (*10*–*15*). This emphasis on group interactions is natural, given how much of life and society is organized around multiway relationships and interactions, such as work collaborations, social activities, volunteer groups, and family ties. However, despite the ubiquity of multiway interactions in social settings, existing homophily measures rely on a graph model of social interactions, which encodes only two-way relationships between individuals. To measure homophily in group interactions, these approaches typically reduce group participation to pairwise relationships based on coparticipation in groups. While this simplifies the analysis, it discards valuable information about the exact size and makeup of groups in which individuals choose to participate.

Here, we present a mathematical framework for measuring homophily in higher-order, multiway interactions that quantifies the extent to which individuals in a certain class participate in groups with varying numbers of in-class and out-class participants. This relies on a hypergraph model for representing group interactions, which generalizes the standard graph model. In the graph setting, homophily can be measured by comparing a graph homophily index against a baseline score (*16*). We generalize this and show that there are many intuitive ways to quantify tendencies toward same-class interactions in group settings. One simplistic example of higher-order homophily is a hypergraph where hyperedges only involve nodes from a single class. As an example, this could represent social interactions where every social group involves only men or only women. However, this matches very few real-world examples and fails to capture other intuitive types of same-class mixing patterns in group interactions. For example, an individual may tend to participate mainly in groups where at least the majority of members are from the same class, even if no groups are completely homogeneous with respect to class. Alternatively, one’s participation in group interactions may increase in proportion to the number of same-class members in the group. Our framework allows us to quantify many of these generalized notions of group homophily and identify patterns in group interactions that cannot be captured by existing graph measures. At the same time, we prove fundamental combinatorial limits on the extent to which these generalized notions of same-class group mixing patterns can be exhibited in practice. In particular, we prove that certain seemingly natural approaches for generalizing graph measures of homophily to the hypergraph setting are overly restrictive and cannot be satisfied by any hypergraph because of combinatorial impossibilities that are independent of human preferences and choices.

Our framework provides a very general way to define and measure higher-order homophily, and our combinatorial impossibilities shed light on empirical observations that would otherwise be hard to explain or easy to misinterpret. For example, our framework captures higher-order homophily present in group interactions defined by legislative bill cosponsorship among U.S. members of congress. One intuitive and expected observation captured by our framework is a higher-than-random tendency for members of congress to cosponsor bills that are mostly (even if not exclusively) cosponsored by members of their same political party. The deeper and less obvious insight revealed by our framework is that for both parties to simultaneously exhibit this behavior, there must be a substantial number of members from each party that are willing to cosponsor bills even when their party is in the minority. Contrary to what a naive understanding of group homophily might suggest, both political parties must exhibit much higher tendencies to participate in bills where their party is overwhelmingly outnumbered, in comparison with bills where their party actually has a slight majority. Our combinatorial impossibility results are key to understanding and interpreting these empirical results. Without them, it would be tempting to conclude that only weak notions of group homophily are satisfied in legislative bill cosponsorship. However, our theoretical results reveal that group homophily is strongly exhibited in this setting. Outside of unrealistic extreme cases where nearly all group interactions are perfectly homogeneous, stronger notions of group homophily are combinatorially impossible.

We similarly use our framework to reveal empirical differences in coauthorship patterns between men and women in academic publishing. These should be interpreted and understood in light of our theoretical results, as many of these differences are due to combinatorial constraints that must be satisfied independent of social factors. Finally, our framework allows us to uncover meaningful patterns in group interactions that cannot be detected by graph homophily measures. As an example, we use our framework to reveal different gender distribution patterns in group pictures, depending on picture context and group size, which are overlooked by graph measures.

## GROUP AFFINITIES AND HYPERGRAPH HOMOPHILY

**Fig. 1.**
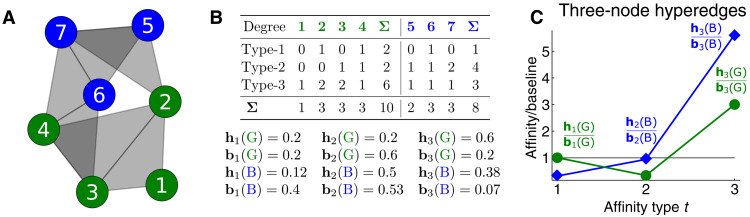
Hypergraph affinity and ratio scores for a small hypergraph. (**A**) An example of a set of size 3 group interactions between two classes, modeled by a small hypergraph. Each triangle in the figure indicates a three-way group interaction, and color indicates node class (green or blue). (**B**) Degrees, affinity scores, and baseline scores for the hypergraph. A node’s type-*t* degree is the number of groups it belongs to in which exactly *t* nodes are from its class. The type-*t* degree for an entire class is the sum of type-*t* degrees across all nodes in the class (Σ columns in the table). The type-*t* affinity for a class *X*, denoted by **h***_t_*(*X*), is the ratio between the class’s type-*t* degree and its total degree (the sum of type-*t* degrees for all *t*). The type-*t* baseline score **b***_t_*(*X*) is the probability that a node joins a type-*t* group if other nodes are selected uniformly at random. (**C**) The ratios of affinity to baseline (ratio scores) summarize the overall group participation rates for both types of nodes.

To introduce our framework, we consider a hypergraph *H* = (*V*, *E*) (Fig. 1A), where the node set *V* represents individuals in a population (e.g., students in a school, researchers in academia, or employees at a company). Each hyperedge *e* ∈ *E* represents a group interaction among members of the population. The set *X* ⊆ *V* indicates a set of individuals with the same class label (e.g., gender or political affiliation). We would like to quantify the extent to which individuals in this class tend to interact and connect with one another in group settings. For our mathematical formulation, we can treat *H* = (*V*, *E*) as a *k*-uniform hypergraph, meaning that each hyperedge represents a multiway relationship among exactly *k* nodes. In practice, the same measures can be applied to each group size *k* separately to analyze homophily patterns that are exhibited in different ways across different groups sizes.

### Measuring group affinities

To measure how class label affects group interactions of a fixed size *k*, we define for each positive integer *t* ∈ [*k*] = {1,2, …, *k*} a type-*t* affinity score, summarizing the extent to which individuals in class *X* participate in groups where exactly *t* group members are in class *X* (Fig. 1B). To define the affinity score, we first define the total degree of a node *v*, denoted by *d*(*v*), to be the number of groups it participates in. Its type-*t* degree, denoted by *d_t_*(*v*), is the number of these groups with exactly *t* members from *v*’s class, including *v* itself. The class degree *D*(*X*) is the sum of total degrees across all nodes in *X*, and the class type-*t* degree *D_t_*(*X*) is the sum of individual type-*t* degrees. The ratio of these values defines the type-*t* affinity score

$$h_{t}(X)=\frac{D_{t}(X)}{D(X)}=\frac{\sum_{v\in X}d_{t}(v)}{\sum_{v\in X}d(v)}$$ (1)

When *k* = *t* = 2, this ratio is the well-studied homophily index of a graph (*16*), the fraction of same-class friendships for class *X*. This index can be statistically interpreted as the maximum likelihood estimate for a certain homophily parameter when a logistic binomial model is applied to the degree data. An analogous result is also true for our more general hypergraph affinity score (see the Supplementary Materials).

### Baseline scores for group affinities

**Fig. 2.**
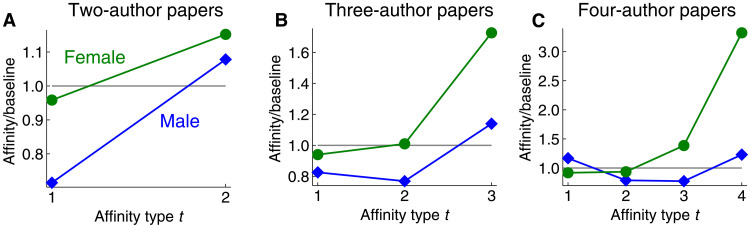
Ratio scores with respect to gender for groups defined by coauthorship in computer science publications. (**A**) Collaborating with same-gender coauthors on two-person papers is more likely than expected by chance, as seen by ratio scores higher than 1. (**B** and **C**) For three- and four-author papers, the ratio score curves are substantially different for men and women. Female authors exhibit monotonically increasing scores, whereas male authors do not. Our theoretical results show that many of these differences are due simply to combinatorial constraints on hypergraph affinity scores. If we reduce the set of two- to four-author papers to pairwise coauthorships and apply graph-based measures, then women and men have graph homophily indices of 0.261 and 0.828, respectively. These scores are higher than group proportions of 0.215 and 0.785 and therefore reveal some level of gender homophily. Similar graph homophily indices are obtained if we also include papers with more authors. However, this provides less information than knowing the full range of hypergraph affinity scores and fails to uncover the nuanced differences in coauthorship patterns between men and women.

To determine whether an affinity score **h***_t_*(*X*) is meaningfully high or low, we compare it against a baseline score **b***_t_*(*X*) representing a null probability for type-*t* interactions. If **h***_t_*(*X*) > **b***_t_*(*X*), then this will indicate that type-*t* group interactions are overexpressed for class *X*. This is analogous to the notion of inbreeding homophily in traditional social network analysis—the tendency for individuals to connect with other similar individuals more than would be expected by chance (*12*, *17*). Given a set of baseline scores, one way to summarize the group participation for a class *X* is to plot a sequence of ratio scores $\frac{h_{t}(X)}{b_{t}(X)}$ for *t* ≤ *k* (Fig. 2C). Ratio scores near one indicate that the class distribution in group interactions is roughly what would be expected at random.

The standard baseline score that we consider (and which we use in Fig. 2C) is the probability that a class *X* node joins a group where *t* members are from class *X*, if *k* − 1 other nodes are selected uniformly at random. Formally, this is given by

$${\hat{b}}_{t}(X)=\frac{\left(\begin{array}{c} |X|-1\\ t-1 \end{array}\right)\left(\begin{array}{c} n-|X|\\ k-t \end{array}\right)}{\left(\begin{array}{c} n-1\\ k-1 \end{array}\right)}$$ (2)

where *n* = *|V|* is the number of nodes in the hypergraph. In the graph setting (*k* = 2), the homophily index **h**_2_(*X*) is typically compared against* α_x_=|X|/n*, the proportion of nodes in class *X* (*16*). Observe that this is simply an asymptotic version of the standard baseline score ${\hat{b}}_{2}(X)=(|X|-1)/(n-1)$. In practice, it is often useful to use asymptotic baseline scores for general *k* and *t* by fixing the class proportion *α_x_=|X|/n* ** and computing $\lim_{n\to \infty }{\hat{b}}_{t}(X)$.

We provide two additional intuitive interpretations for this standard baseline score. The first is that baseline scores correspond to affinity scores for a complete *k*-uniform hypergraph. The second is that generating random hyperedges without regard for node class produces a hypergraph whose ratio scores asymptotically converge to 1. We prove these results in the Supplementary Materials.

**Proposition 1.** *Let* $\$H_{k,n}^{*}=(V,E)\$$ *be the complete k-uniform hypergraph on n nodes. The type-t affinity score for class X* ⊆ *V equal the type-t baseline score in* Eq. 2.

**Proposition 2.** *Fix any p* ∈ (0,1) *and a positive integer k and let H* = (*V*, *E*) *be a random hypergraph on n nodes that is formed by turning each k-tuple of nodes in V into a hyperedge with probability p. As n* → ∞*, the ratio scores for a class X* ⊆ *V with* *|X|* = Θ(*n*) *converge in probability to 1*.

While the baseline scores in Eq. 2 are a natural choice for a number reasons, it is also possible to define and consider baselines corresponding to other null probabilities for affinity scores. The combinatorial impossibility results that we prove later will apply to a more general class of realizable scores. A set of baseline scores {**b***_t_*(*X*) : *t* ∈ [*k*]} is realizable if they are all positive and there exists a hypergraph whose affinity scores are exactly these baselines scores. Proposition 1 indicates that the scores in Eq. 2 are realizable.

### Defining higher-order homophily

Informally, homophily is the tendency for individuals in one class to disproportionately interact and connect with other individuals in the same class. In a graph setting, this can be measured by checking whether nodes tend to form links with other same-class nodes more often than random. We consider three natural ways to extend this to the hypergraph setting.

One simplistic way to check for group homophily is to see whether a class has a higher-than-baseline affinity for group interactions that only involve members of their class. Formally, this means that **h***_t_*(*X*) > **b***_t_*(*X*) for *t* = *k*. We refer to this as simple homophily. This captures one valid notion of hypergraph homophily but is very restrictive and fails to capture other intuitive types of same-class mixing patterns. This includes high affinities for groups where at least a majority of members are from the same class or group participation levels that increase in proportion to the number of same-class members in a group.

To capture more nuanced notions of group homophily, we say that class *X* exhibits order*-j* majority homophily if the top *j* affinity scores for this class are higher than baseline, i.e., **h**_*k*−*j*+1_(*X*) > **b**_*k*−*j*+1_(*X*), **h**_*k*−*j*+2_(*X*) > **b**_*k*−*j*+2_(*X*), … , **h***_k_*(*X*) > **b***_k_*(*X*). Simple homophily is the special case of order-*1* majority homophily, while larger values of *j* capture generalized and stronger notions of homophily. We say that a class has order*-j* monotonic homophily if each of the top *j* ratio scores is larger than the ratio score that comes before it. Formally, this means **h***_t_*(*X*)/**b***_t_*(*X*) > **h**_*t*−1_(*X*)/**b**_*t*−1_(*X*) for *t* ≥ *k* − *j* + 1. In other words, ratio scores from **h**_*k*−*j*_(*X*)/**b**_*k*−*j*_(*X*) to **h***_k_*(*X*)/**b***_k_*(*X*) are strictly increasing. Finally, we say that the majority homophily index (MaHI) of a class *X* is the maximum *j* such that order-*j* majority homophily holds, and the monotonic homophily index (MoHI) is the maximum *j* such that order-*j* monotonic homophily holds.

### Strict notions of higher-order homophily

We also consider two basic measures for majority and monotonic group participation that seem intuitive at first, but which we will prove are much more restrictive than they appear. We say that class *X* exhibits strict majority homophily if the class has higher than random affinities for groups where they constitute a majority, i.e., **h***_t_*(*X*) > **b***_t_*(*X*) when *t* > *k* − *t*. This is equivalent to order-(⌈*k*/2⌉) majority homophily. Similarly, class *X* exhibits strict monotonic homophily if, for groups where *X* constitutes a majority, its ratio scores are strictly increasing as the number of class *X* members grows, i.e., **h***_t_*(*X*)/**b***_t_*(*X*) > **h**_*t*−1_(*X*)/**b**_*t*−1_(*X*) when *t* > *k* − *t*. This is equivalent to order-(⌈*k*/2⌉) monotonic homophily. For groups of size two, the notions of simple homophily, strict majority homophily, and strict monotonic homophily all reduce to checking whether the homophily index of a graph is higher than the relative class size. This is a standard way of checking for graph homophily (*16*). In graph-based analysis, it is typical for multiple node classes in a social network to exhibit homophily at the same time. A natural question is whether this observation generalizes to the hypergraph setting.

### Gender affinities in coauthorship data

As a first example, we measure hypergraph affinity scores with respect to gender in academic collaborations, where nodes represent researchers and each hyperedge indicates coauthorship on a paper published at a computer science conference. Our framework reveals differences in coauthor patterns for men and women (Fig. 2). Both men and women have overexpressed tendencies for being authors on papers that only involve authors of their same gender. In other words, for collaborations of size two to four, both genders exhibit simple homophily.

For two-author papers, both genders exhibit strict monotonic homophily and strict majority homophily, as these coincide with simple homophily. For three- and four-author papers, women exhibit both strict majority and strict monotonic homophily, but men do not exhibit either. These definitions seem to capture intuitive higher-order notions of homophily, and if we restrict them to the graph setting, then we recover existing notions of homophily that are often satisfied by multiple classes at once. How, then, can we explain the differences between men and women in this dataset? It is tempting to wonder whether these differences are purely due to social factors. In other contexts, can we expect both men and women to exhibit strict monotonic and strict majority homophily? Our main theoretical results will show, perhaps unexpectedly, that this is impossible for any dataset and that many of the differences that we see between men and women in our coauthorship results must exist simply because of combinatorial inevitabilities. It is not immediately clear why this should be, given that men and women have separate affinity scores as well as separate baseline scores. These combinatorial limits provide a deeper understanding of how higher-order homophily can be manifested in practice. This also highlights pitfalls and misunderstandings to avoid when drawing conclusions about the presence or level of group homophily in different contexts.

## IMPOSSIBILITY RESULTS FOR STRICT HOMOPHILY

Our main theoretical results highlight combinatorial constraints that govern higher-order mixing patterns in hypergraphs. These are easily overlooked but are crucial for properly defining and understanding higher-order homophily. This also reveals a fundamental difference between measuring homophily in group settings and measuring homophily in graphs, as these impossibilities do not apply to the graph setting. Although strict monotonic and strict majority homophily seem to capture intuitive notions of same-class mixing patterns, we show that it is combinatorially impossible for two classes to simultaneously exhibit either of these types of homophily in the hypergraph setting (subject to a small additional constraint if groups have an even number of members). In other words, even if all individuals preferred to participate in group interactions that are monotonically or majority homophilous with respect to their class, this cannot be accomplished.

We formalize our results as a set of combinatorial impossibilities for two-class, *k*-uniform hypergraphs. Formally, a hypergraph *H* = (*V*, *E*) is a two-class, *k*-uniform hypergraph if ∣*e* ∣ = *k* for every *e* ∈ *E*, and there exist two node classes {*A*, *B*} such that *V* = *A* ∪ B and *A* ∩ *B* = ∅. Although our theorems focus on this family of hypergraphs, our framework and results have important implications for understanding homophily in general group settings. When groups vary in size, our results can be applied to each group size separately, to understand which behaviors are possible or impossible in each case. We take this approach when measuring affinity scores on real datasets involving group interactions of varying size *k*. For a hypergraph with more than two node labels, our results imply combinatorial impossibilities for an arbitrary class *A* = *X* ⊆ *V* relative to the collective behavior of all other classes, joined by a single “out class” label *B* = *V *\*X*.

### Equivalent characterization of affinity scores

In proving our impossibility results for a *k*-uniform hypergraph with classes *A* and *B*, it will be convenient to categorize hyperedges based on the number of nodes from each class. Although type-*t* degrees and type-*t* affinity scores are defined relative to a given class *X*, we define hyperedge types in an absolute sense: For *j* ∈ {0, 1, 2, …, *k*}, a hyperedge *e* ∈ *E* is of type-*j* if it contains exactly *j* nodes specifically from class *A*. We denote the number of type-*j* hyperedges by *m_j_*. This allows us to write type-*t* affinity scores in terms of absolute hyperedge counts {*m*_0_, *m*_1_, …, *m_k_*}.

The type-*t* affinities for *A* and *B* are then

$$h_{t}(A)=\frac{tm_{t}}{\sum_{i=1}^{k}im_{i}}\;\mathrm{and}\;h_{t}(B)=\frac{tm_{k-t}}{\sum_{i=1}^{k}im_{k-i}}$$ (3)

Observe that in the numerator of **h***_t_*(*A*), we scale *m_t_* by *t* to account for the fact that each type-*t* hyperedge involves *t* nodes from class *A* and therefore contributes to the degree of *t* different nodes in class *A*. Meanwhile, type–(*k* − *t*) hyperedges involve *t* nodes from class *B*, leading to the expression for **h***_t_*(*B*).

### Impossibility results for strict monotonic homophily

We begin with an impossibility result for monotonic homophily. This has a comparatively simple proof that relies on considering two contradictory inequalities that result from assuming two classes exhibit homophily. We separate our results based on whether *k* is odd or even.

**Theorem 3.** *Let H be a two-class, k*-*uniform hypergraph and* {***b****_i_*(*X*) : *i* ∈ [*k*], *X* ∈ {*A*, *B*}} *be realizable baseline scores. For odd k, it is impossible for both classes to exhibit strict monotonic homophily.*

*Proof.* If both classes *A* and *B* exhibit strict monotonic homophily, then the following two sequences of inequalities hold

$$\text{Class A}:\frac{h_{k}(A)}{b_{k}(A)}>\frac{h_{k-1}(A)}{b_{k-1}(A)}>\cdots >\frac{h_{r}(A)}{b_{r}(A)}>\frac{h_{r-1}(A)}{b_{r-1}(A)}$$ (4)

$$\text{Class B}:\frac{h_{k}(B)}{b_{k}(B)}>\frac{h_{k-1}(B)}{b_{k-1}(B)}>\cdots >\frac{h_{r}(B)}{b_{r}(B)}>\frac{h_{r-1}(B)}{b_{r-1}(B)}$$ (5)

where *r* = (*k* + 1)/2.

Using the characterization of affinity scores given in Eq. 3, the last inequality in each of Eqs. 4 and 5 can be rearranged as follows

$$\frac{h_{r}(A)}{b_{r}(A)}>\frac{h_{r-1}(A)}{b_{r-1}(A)}⟺\frac{rm_{r}}{b_{r}(A)}>\frac{(r-1)m_{r-1}}{b_{r-1}(A)}$$ (6)

$$\frac{h_{r}(B)}{b_{r}(B)}>\frac{h_{r-1}(B)}{b_{r-1}(B)}⟺\frac{rm_{r-1}}{b_{r}(B)}>\frac{(r-1)m_{r}}{b_{r-1}(B)}$$ (7)

Above, we have used the observation that *m*_*k*−*r*_ = *m*_*r*−1_ and *m*_*k*−(*r*−1)_ = *m_r_* to write both inequalities in terms of *m_r_* and *m*_*r*−1_.

Because the baseline scores are realizable, there exists some two-class *k*-uniform hypergraph *G* whose affinity scores equal the baseline scores. Letting *M_t_* denote the number of type-*t* hyperedges in *G*, we can write the baseline scores as

$$b_{r}(A)=\frac{rM_{r}}{D_{A}},b_{r-1}(A)=\frac{(r-1)M_{r-1}}{D_{A}},$$

$$b_{r}(B)=\frac{rM_{r-1}}{D_{B}},b_{r-1}(B)=\frac{(r-1)M_{r}}{D_{B}},$$

where $D_{A}=\sum_{i=1}^{k}iM_{i}$ and $D_{B}=\sum_{i=1}^{k}iM_{k-i}$. Applying a few steps of algebra to the inequality on the right of Eq. 6 shows that if *A* exhibits monotonic homophily, then

$$\frac{rm_{r}}{b_{r}(A)}>\frac{(r-1)m_{r-1}}{b_{r-1}(A)}⟺\frac{m_{r}}{M_{r}}>\frac{m_{r-1}}{M_{r-1}}$$

Meanwhile, the inequality in Eq. 7 implies the exact opposite

$$\frac{rm_{r-1}}{b_{r}(B)}>\frac{(r-1)m_{r}}{b_{r-1}(B)}⟺\frac{m_{r-1}}{M_{r-1}}>\frac{m_{r}}{M_{r}}$$

Thus, assuming both classes exhibit strict monotonic homophily leads to a contradiction.

Strict monotonic homophily is possible for two classes at once if *k* is even. This can happen, for example, by starting with a complete *k*-uniform hypergraph and deleting all type-*k*/2 hyperedges, if we are specifically considering standard baseline scores from Eq. 2. However, an analogous impossibility result holds if we add one extra assumption. The proof follows the same strategy as the proof of Theorem 3, after adding an extra inequality for one class.

**Theorem 4.** *Let H be a two-class, k-uniform hypergraph and {***b****_i_*(X) : i ∈ [k], X ∈ {*A*, *B*}} be realizable baseline scores. If k is even, then it is impossible for both classes to satisfy strict monotonic homophily if additionally $\frac{h_{\ell }(X)}{b_{\ell }(X)}>\frac{h_{\ell -1}(X)}{b_{\ell -1}(X)}$ for one class X ∈ {*A*, *B*}, where 𝓁 = k/2.*

Theorems 3 and 4 lead to other impossibility results as direct corollaries. Our definition of strict monotonic homophily for a class *X* is defined specifically for groups where *X* is in the majority. If we remove this restriction and consider a strict notion of monotonic homophily that requires **h***_t_*(*X*)/**b***_t_*(*X*) > **h**_*t*−1_(*X*)/**b**_*t*−1_(*X*) for all *t* ≤ *k*, then for every *k* > 2, this is impossible for two classes simultaneously whether or not *k* is odd. We also see from the proof of Theorem 3 that a contradiction results from assuming both classes have increasing ratio scores when going from type–(*r* −1) to type-*r* affinities. Thus, any notion of homophily involving this assumption is impossible for two classes at once, regardless of what happens with other ratio scores.

### Impossibility results for strict majority homophily

Next, we turn to extremal results for majority homophily.

**Theorem 5.** *Let H* = (*V*, *E*) *be a two-class k-uniform hypergraph and* {***b****_i_*(*X*) : *i* ∈ [*k*], *X* ∈ {*A*, *B*}} *be realizable baseline scores*

• *If k is odd, then it is impossible for both classes to simultaneously exhibit strict majority homophily.*

*• If k is even, then it is impossible for both classes to exhibit strict majority homophily if additionally* **h**_*k*/2_(*X*) > **b**_*k*/2_(*X*) *for one of the classes X ∈ {*A*, *B*}.*

Although our results for strict majority homophily closely mirror our results for strict monotonic homophily, Theorem 5 is significantly more challenging to show and requires a different and more in-depth proof technique. A full proof is provided in the Supplementary Materials. We provide a detailed proof sketch of the result for odd values of *k*. The same overall strategy yields the result for even *k*.

For odd *k* and *r* = (*k* + 1)/2, assuming that both classes exhibit strict majority homophily is equivalent to satisfying two sets of inequalities

$$h_{t}(A)>b_{t}(A)\;\mathrm{for}\;t>k-t$$ (8)

$$h_{t}(B)>b_{t}(B)\;\mathrm{for}\;t>k-t.$$ (9)

Using the characterization of affinity scores given in Eq. 3, we can rearrange these inequalities to yield equivalent inequalities in terms of typed hyperedge counts {*m*_0_, *m*_1_, …, *m_k_*}:

$$m_{t}>\frac{b_{t}(A)\sum_{i\neq t}im_{i}}{t\cdot [1-b_{t}(A)]},\mathrm{for}\;t=r,r+1,\ldots ,k$$ (10)

$$m_{s}>\frac{b_{k-s}(B)\sum_{i\neq k-s}im_{k-i}}{\left(k-s)\cdot [1-b_{k-s}(B)\right]},\mathrm{for}\;s=0,1,\ldots ,r-1.$$ (11)

Our aim is to show that all of the above inequalities cannot hold simultaneously.

It is important to note that although every hyperedge type is bounded below by one of the inequalities in Eqs. 10 and 11, this does not immediately imply any contradiction. This is because half of the hyperedge types are bounded below in terms of baseline scores for class *A*, while the other half are bounded below in terms of baseline scores for class *B*. Baseline scores for *A* and *B* can differ substantially if these classes differ in size, even if we consider the very special case of standard scores given by Eq. 2. We ultimately show that these inequalities cannot be satisfied simultaneously for any set of realizable baseline scores by analytically finding solutions to a linear program (LP) encoding the maximum amount of homophily that can be satisfied by two classes at once. Because of the complexity of this proof, we begin by considering other approaches that seem simple and natural at first but ultimately fail to prove the main result.

We first of all note that it is simple to show that hypergraph affinity scores for a single class *X* cannot all be simultaneously above baseline, i.e., **h***_t_*(*X*) > **b***_t_*(*X*) for all *t* ∈ [*k*]. Because hyperedge affinity scores sum to one and baseline scores do as well, summing both sides of the inequality for all *t* leads to an immediate contradiction. This argument does not apply if we assume two classes exhibit strict majority homophily, since, in this case, only some types of interactions are above the baseline for class *A*, while other types of interactions are above baseline for class *B*. A next approach for trying to prove Theorem 5 is to sum up the left and right hand sides of the hyperedge inequalities in Eqs. 10 and 11 and see if this leads to a contradiction. However, this also does not work. For example, let *k* = 3 and assume that we use standard baseline scores from Eq. 2. If classes are equal in size, then ${\hat{b}}_{t}={\hat{b}}_{t}(A)={\hat{b}}_{t}(B)$ for *t* ∈ {1,2,3}. Summing both sides of inequalities in Eqs. 10 and 11 leads to a new inequality

$$3(m_{0}+m_{3})+2(m_{1}+m_{2})>3(m_{0}+m_{1}+m_{2}+m_{3})({\hat{b}}_{2}+{\hat{b}}_{3})$$

This is satisfied by any hypergraph where *m*_1_ = *m*_2_ = 0, so it does not provide the contradiction that we are looking for.

The proof of Theorem 3 shows that a subset of the strict monotonic homophily inequalities (in fact, just two of them) leads to a contradiction. Therefore, another natural strategy for trying to prove Theorem 5 is to see whether a subset of the inequalities given by Eqs. 10 and 11 contradict each other. In the Supplementary Materials, we prove the following result, which rules out this possibility.

**Proposition 6.** *If any inequality from* Eqs. 10 *and* 11 *is discarded, then it is possible to construct a two-class k-uniform hypergraph satisfying the remaining inequalities.*

This means that any strategy similar to the proof for Theorem 3 will fail for Theorem 5. Instead, any proof for Theorem 5 will need to incorporate every one of the inequalities in Eqs. 10 and 11 if we are to show that strict majority homophily is impossible.

#### Capturing homophily limits via linear programming

Having ruled out simpler strategies for proving Theorem 5, we now outline a linear programming framework for checking the maximum amount of homophily that can be exhibited in a set of group interactions, subject to different constraints on higher-order affinity scores. This first of all provides a general framework for numerically checking whether different extremal notions of higher-order homophily can be satisfied or not. We will also show how to use analytical solutions and linear programming duality to fully prove Theorem 5.

We specifically consider an LP that encodes the maximum amount of majority homophily that can be satisfied by two classes *A* and *B* simultaneously in a *k*-uniform two-class hypergraph. This LP is given by

$$\begin{array}{rl} & \max\;\gamma \\ & s.t.\;\sum_{i=0}^{k}x_{i}=1\\ & \;\;t\cdot x_{t}-b_{t}(A)\cdot \sum_{i=0}^{k}i\cdot x_{i}\geq \gamma \;\;\mathrm{for}\;t\in \{r,\ldots ,k\}\\ & \;\;\;t\cdot x_{k-t}-b_{t}(B)\cdot \sum_{i=0}^{k}i\cdot x_{k-i}\geq \gamma \;\mathrm{for}\;t\in \{r,\ldots ,k\}\\ & \;x_{i}\geq 0\;\;\;\;\;\;\;\;\;\mathrm{for}\;i\in \{0\}\cup [k] \end{array}$$ (12)

In this LP, there is a variable *x_i_* ≥ 0 for each type of hyperedge in some hypergraph. More specifically, the constraint $\sum_{i=0}^{k}x_{i}=1$ encodes the fact that *x_i_* represents the proportion of hyperedges that are of type-*i*. The constraint

$$t\cdot x_{t}-b_{t}(A)\cdot \sum_{i=1}^{k}i\cdot x_{i}\geq \gamma$$

can be rearranged into the inequality

$$\frac{t\cdot x_{t}}{\sum_{i=1}^{k}i\cdot x_{i}}\geq b_{t}(A)+\frac{\gamma }{\sum_{i=1}^{k}i\cdot x_{i}}$$

This constrains the type-*t* affinity score for class *A* to be larger than its baseline score by at least an additive term $\gamma /\sum_{i=1}^{k}i\cdot x_{i}$, which will be positive if and only if γ is positive. The second set of constraints encodes similar bounds for the affinity scores of class *B*. A feasible solution with γ = 0 can always be achieved if the *x_i_* variables represent hyperedge counts for a hypergraph whose affinity scores are equal to the realizable baseline scores. We prove the following results in the Supplementary Materials.

**Lemma 7.** Let γ^*^ be the optimal solution to the LP in Eq. 12. There exists a two-class k-uniform hypergraph where both classes exhibit strict majority homophily if and only if γ^*^ > 0.

Given this result, we can check numerically whether strict majority homophily can hold for two classes, as long as we are given a fixed set of baseline scores and a fixed *k*. However, numerical solutions do not provide a full proof of our result for general baseline scores and arbitrary *k*. To prove our theorem, we consider the dual of the LP in Eq. 12, which is given by

$$\begin{array}{rl} & \min\;\alpha \\ & s.t.\;\sum_{t=r}^{k}y_{A,t}+y_{B,t}\geq 1\\ & \;-{iy}_{B,i}+(k-i)\sum_{j=r}^{k}y_{A,j}b_{j}(A)+i\sum_{j=r}^{k}y_{B,j}b_{j}(B)\\ & \;\;+\alpha \geq 1\;\mathrm{for}\;i\in \{0\}\cup [k]\\ & y_{A,t}\geq 0\;\;\;\mathrm{for}\;t\in \{r,\ldots ,k\}\\ & y_{B,t}\geq 0\;\;\;\mathrm{for}\;t\in \{r,\ldots ,k\} \end{array}$$ (13)

We prove a key result regarding a set of feasible variables for the dual LP.

**Lemma 8.** *For an odd integer k and r* = (*k* + 1)/2, *define* $\delta =2k\sum_{t=r}^{k}\frac{1}{t}$ *and consider the following set of dual variables*

$$\begin{array}{rl} & \;\alpha =0\\ & y_{B,k}=\frac{2}{\delta }\cdot \frac{\sum_{i=r}^{k}\left(\frac{k}{i}-1\right)b_{i}(B)}{1-\sum_{i=r}^{k}\left(2-\frac{k}{i}\right)b_{i}(B)}\\ & y_{B,t}=\frac{2}{\delta }\left(\frac{k}{t}-1\right)+\left(2-\frac{k}{t}\right)y_{B,k}\;for\;t\in \{r,\cdots ,k-1\}\\ & y_{A,t}=\frac{2k}{\delta t}-y_{B,t}\;\;for\;t\in \{r,\cdots ,k\} \end{array}$$

*If* $Y=\sum_{t=r}^{k}y_{A,t}+y_{B,t}$, *then the set of normalized dual variables defined by* ${\tilde{y}}_{X,t}=y_{X,t}/Y$ *for X* ∈ {*A*, *B*} *and t* ∈ {*r*, …, *k*} *is feasible for the dual LP in* Eq. 13.

The full proof of this lemma, provided in the Supplementary Materials, is quite involved and relies on the fact that the baseline scores are realizable. Once the result is proven, it immediately implies our impossibility result for odd *k*. By linear programming duality, any feasible solution for the dual LP provides an upper bound on the solution to the primal LP. Because the dual variables that we provide in Lemma 8 come with an objective score of α = 0, we know the optimal solution to the primal LP is also 0. By Lemma 7, strict majority homophily must be impossible to satisfy for both classes *A* and *B* at once if *k* is odd. For even *k*, the impossibility result in Theorem 5 can be shown by adding one more constraint to the primal LP and providing an analytical solution to the new dual LP, similar to Lemma 8.

While our linear programming framework does not constitute the only way to prove Theorem 5, it is a useful approach for capturing extremal limits of higher-order homophily beyond this specific result and its proof. The linear constraints encoding bounds on different affinity scores can be easily altered to quickly check the feasibility of other notions of homophily. For example, one could quickly check whether the top *i* ratio scores can all be above a certain fixed threshold for two node classes at once, for different values of *i*. This LP framework can also be used more broadly as a proof technique for other theoretical results. Our proof of Proposition 6 in the Supplementary Materials makes use of the LP formulation in Eq. 12 and Lemma 7. We can also use an alternative LP and LP duality proof to prove Theorems 3 and 4. In this case, and unlike Lemma 8, most of the optimal dual variables for an LP encoding strict monotonic homophily end up being zero, except for the dual variables associated with the contradictory constraints in inequalities in Eqs. 6 and 7. This simplifies the proof for monotonic homophily and again indicates that the result for monotonic homophily is simpler to show than the corresponding result for majority homophily.

### Alternative affinity scores and normalizations

Several slightly different measures of graph homophily have been considered in previous research (*16*–*19*), and in the same way, there is more than one way to quantify homophily in the hypergraph setting. We additionally consider the following alternative hypergraph affinity scores

$${\tilde{h}}_{t}(A)=\frac{m_{t}}{\sum_{i=1}^{k}m_{i}}\;\mathrm{and}\;{\tilde{h}}_{t}(B)=\frac{m_{k-t}}{\sum_{i=1}^{k}m_{k-i}}$$ (14)

Unlike the affinity scores in Eq. 3, which are equivalent to our original definition in Eq. 1, these scores directly depend on the proportion of different hyperedge types. This is another natural approach for quantifying an entire class’s group interaction patterns. In the Supplementary Materials, we derive matching combinatorial impossibilities for these alternative scores, showing that our main results persist across various notions of group affinities. We primarily focus on the affinity score in Eq. 1, defined by ratios of typed degrees, as this directly generalizes an existing notion of a graph homophily index (*16*). This focus on node degrees is also shared by other closely related measures of graph homophily (*17*, *18*) and provides a way to capture the average experience or behavior of an individual in a certain node class.

There is also more than one approach to measuring how much a graph homophily index deviates from a null model. One useful normalization in the graph setting is to consider how much a graph homophily index deviates from its baseline, relative to the maximum amount that it could deviate from baseline (*17*, *18*). We can incorporate this notion into our hypergraph framework by defining the following type-*t* normalized bias score

$$f_{t}(X)=\{\begin{array}{cc} \frac{h_{t}(X)-b_{t}(X)}{1-b_{t}(X)} & \mathrm{if}\;h_{t}(X)\geq b_{t}(X)\\ \frac{h_{t}(X)-b_{t}(X)}{b_{t}(X)} & \mathrm{if}\;h_{t}(X)<b_{t}(X) \end{array}$$ (15)

The value **h***_t_*(*X*) − **b***_t_*(*X*) is the bias that class *X* has for type-*t* interactions, and **f***_t_*(*X*) normalizes this by the maximum possible bias. If this affinity is overexpressed [**h***_t_*(*X*) > **b***_t_*(*X*)], then it has a positive bias and the maximum bias is achieved when **h***_t_*(*X*) = 1. When the affinity is underexpressed, the maximum deviation from baseline is when **h***_t_*(*X*) = 0, and we therefore normalize by **b***_t_*(*X*). The normalized bias score conveys useful information both in terms of its sign and magnitude. The sign indicates whether an affinity has a positive or negative bias, and the magnitude is always a value between 0 and 1 that indicates how close it comes to its maximum bias. While the magnitude of a ratio score **h***_t_*(*X*)/**b***_t_*(*X*) may depend on the hypergraph size, the fact that **f***_t_*(*X*) is always between −1 and 1 makes it a particularly useful score to use when comparing notions of homophily across different datasets. In our empirical results, we will often consider normalized bias scores in addition to raw affinity scores and ratio scores. Finally, in the Supplementary Materials, we show that Theorems 3 to 5 immediately lead to analogous impossibility results for normalized bias scores as well. We see therefore that our main impossibility results persist across a wide range of different notions of higher-order homophily and that our framework easily accommodates different approaches for measuring deviation from a null model.

## EMPIRICAL RESULTS

Our theoretical results reveal that seemingly natural notions of group homophily are overly strict and cannot be exhibited, simply because of combinatorial impossibilities. However, this by no means implies that higher-order homophily cannot be meaningfully measured or exhibited in practice. To the contrary, establishing limits on what is combinatorially possible in group homophily allows us to better interpret and appreciate relaxed notions of majority and monotonic group homophily that do hold in practice despite being very close to the combinatorial limits of group homophily. We specifically apply our framework to study group homophily in legislative bill cosponsorship, online hotel reviewing, shopping trip data, and group picture data. Our framework reveals new insights into the way same-class group mixing patterns are exhibited in these settings and allows us to uncover structure and patterns that are missed when applying graph homophily measures, which only account only for pairwise interactions.

### Political homophily in legislative bill cosponsorship

**Fig. 3.**
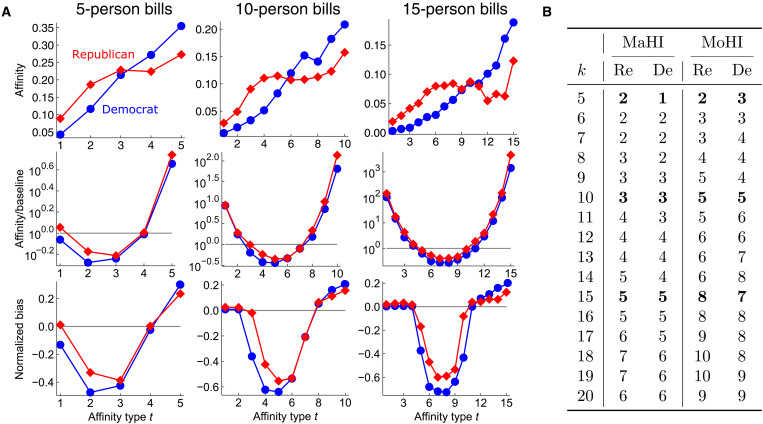
U.S. members of congress (nodes) cosponsoring bills (hyperedges) exhibit certain notions of same-class homophily in terms of political party. (**A**) Affinity scores (top row) increase for Democrats but are relatively flat for Republicans. However, after dividing by baselines (middle row), both classes exhibit bowl-shaped ratio scores that nearly satisfy majority homophily (higher-than-baseline affinities for groups where one’s class is the majority) and monotonic homophily (strictly increasing ratio scores for groups where one’s class is in the majority) without ever violating our theoretical impossibility results. For example, for bills with five cosponsors, Democrats exhibit monotonic homophily, and Republicans almost do as well, except for a slight decrease in scores from *t* = 2 to *t* = 3 (middle row, leftmost). Similar observations hold for bills of other cosponsorship sizes. (**B**) Both Republicans (Re) and Democrats (De) almost always exhibit the highest possible MoHI without violating combinatorial limits (e.g., MoHI of *k*/2 for both parties when *k* is even). Neither class exhibits majority homophily. However, as group size increases, the MaHI (the number of top affinity scores above baseline) increases. Bold entries correspond to plots for bills with 5, 10, and 15 cosponsors. Our results are also robust to perturbations in the data; we obtain nearly identical plots when averaging scores obtained by repeatedly subsampling hyperedges from the dataset (see the Supplementary Materials).

We quantify political homophily (*5*, *20*–*23*) with respect to political party for U.S. members of congress (nodes in a hypergraph), based on groups of congress members formed by cosponsorship of legislative bills (hyperedges) (*24*–*26*). Affinity scores for Democrats strictly increase for most group sizes, although scores are flatter for Republicans (Fig. 3A, top row). However, both classes exhibit similar bowl-shaped ratio curves and normalized bias curves (Fig. 3A, bottom two rows), demonstrating that highly imbalanced groups are highly overexpressed.

Our framework reveals very strong notions of group homophily, at the most extreme limits of what is combinatorially possible. Recall that for even-sized groups, strict monotonic homophily is the most extreme example of what is combinatorially possible for two classes simultaneously, while for odd-sized groups, it lies just beyond the combinatorially feasible boundary. Ratio scores for the two political parties almost perfectly match these extreme combinatorial limits. Both parties satisfy strict monotonic homophily for most even-sized groups, which is reflected in MoHIs of *k*/2 when *k* is even (Fig. 3B). When group size *k* is odd, we typically see one party exhibit strict monotonic homophily [MoHI of (*k* + 1)/2], while the other party just barely fails to satisfy strict majority homophily [MoHI of (*k* − 1)/2]. In other words, for nearly every group size, we observe the maximum level of monotonic homophily that can be exhibited by two classes simultaneously. Across all bill sizes, neither political party exhibits strict majority homophily, but both classes exhibit higher-than-random affinity scores when their political party makes up a large enough majority of bill cosponsors. This is formally captured by MaHIs that steadily increase for each political party as the group size *k* increases.

The bowl-shaped ratio curves and normalized bias curves for Democrats and Republicans also illustrate a point that at first seems counterintuitive but is easily understood in light of our framework. For both parties to exhibit overexpressed affinities for groups where they have a large majority, a substantial number of individuals from each party must be willing to participate in groups where their party is in the minority. Overall, both parties have a higher tendency to participate in cosponsorship groups where they are very outnumbered, compared with participating in groups where they constitute just a slight majority. The normalized bias scores (third row of Fig. 3A) for these slight-majority groups are close to the minimum score of −1, indicating that affinities are almost as far below baseline as they could be. This at first seems to contradict the notion of homophily in group interactions, but our theoretical results explain why this must hold for both parties to satisfy strong notions of group homophily.

The major difference between affinity scores **h***_t_*(*X*) and ratio scores **h***_t_*(*X*)/**b***_t_*(*X*) arises because selecting group members at random from two balanced classes would naturally tend to produce class-balanced groups. In other words, the baseline scores [**b**_1_(*X*), **b**_2_(*X*), …, **b***_k_*(*X*)] will be imbalanced as group size *k* grows, with **b***_t_*(*X*) decreasing as *t* approaches extreme values. Flat or increasing affinity scores for both political parties (Fig. 3A, top row) indicate that the social processes driving bill cosponsorship have overcome the tendency toward class-balanced group interactions that would be expected at random. This reveals another major difference between measuring group homophily and measuring homophily in graphs. For class-balanced graphs, roughly equal affinity scores **h**_1_(*X*) ≈ **h**_2_(*X*) ≈ 0.5 indicate that there is no clear tendency toward in-class or out-class links, i.e., no clear tendency toward homophily.

### Location homophily in trip reviews

**Fig. 4.**
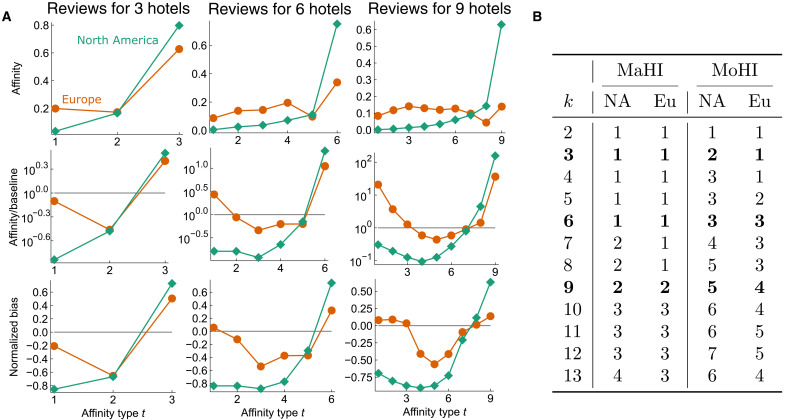
Measures of homophily with respect to location for groups of coreviewed vacation rentals. (**A**) Affinity, ratio, and normalized bias scores on a hypergraph where nodes are hotels, separated into to location classes (North America and Europe), and hyperedges are sets of hotels reviewed by the same user account on Tripadvisor (*27*). Results are comparable to our findings on political homophily in legislative bill cosponsorship. In each case, both classes have monotonic or nearly monotonic ratio scores (second row of plots) for groups where their class is in the majority. (**B**) MoHIs for both North America (NA) and Europe (Eu) are usually at the most extreme limits of what is combinatorially possible. The increasing MaHI shows that classes do tend to exhibit high affinities for groups where their class has a substantial majority, but this is not the case for groups where their class has only a slight majority.

Our framework also applies to hypergraphs that do not encode social interactions. We compute affinity scores for a hypergraph where nodes are hotels on tripadvisor.com from two location classes, North America and Europe, and each hyperedge indicates a group of hotels reviewed by the same user account (Fig. 4A) (*27*). Affinity scores demonstrate intuitive location homophily in review information: Users tend to review hotels that are in the same location. MoHIs for both location classes are at the extreme combinatorial limits of what is possible for two classes simultaneously, across different numbers of reviews. Furthermore, affinities are higher than random for reviewing sets of hotels as long as a substantial majority of the hotels are from the same location (Fig. 4B).

### Product homophily in shopping baskets

**Fig. 5.**
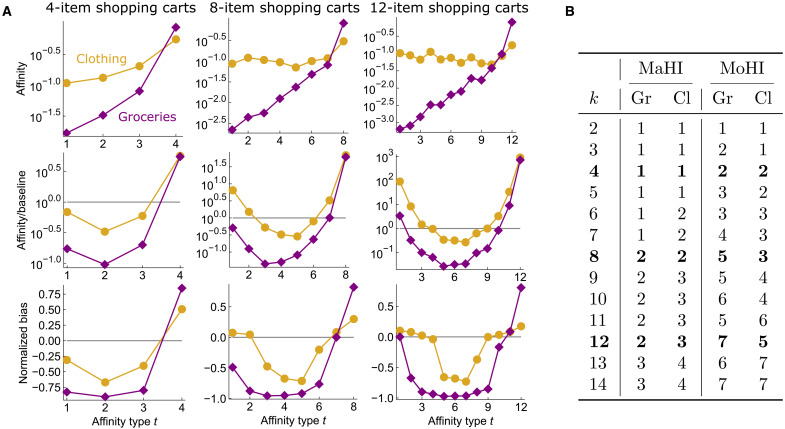
Measures of homophily with respect to product type for groups of copurchased retail products. (**A**) Affinity, ratio, and normalized bias scores for a hypergraph where nodes indicate clothes (Cl) and grocery (Gr) products at Walmart and hyperedges indicate sets of copurchased items during a shopping trip. The bowl-shaped ratio and normalized bias curves for both products indicate that it is typical for shopping trips to primarily focus on one type of product or the other. The fact that affinity scores for grocery products are mostly increasing, while affinity scores for clothing are mostly flat, also matches basic intuition about shopping trips. For example, the relatively small gap between type-*k* and type–(*k* – 1) affinities for clothing is indicative of the fact that when going clothes shopping, it is not uncommon to pick up a needed grocery item while at the store. In contrast, when grocery shopping, it is much less common to additionally pick up a small number of clothing items at the store. This is reflected in the larger gaps between affinity scores and normalized bias scores for groceries. (**B**) The MaHIs grow as shopping basket size increases. MoHIs are also high for both classes.

We also compute affinity scores for a hypergraph derived from a dataset on Walmart shopping trips (*28*). Each node is a product, and hyperedges represent sets of copurchased products (i.e., shopping baskets). Each product comes with a store department label. The labels “Food, Household & Pets” and “Clothing, Shoes & Accessories” make up over half of the original dataset, indicating that a large proportion of shopping purchases can broadly be categorized as clothes purchases or grocery purchases. We consider the two-class hypergraph obtained by restricting to products with these two labels and compute affinity, ratio, and normalized bias scores. Similar to our empirical results on congress bills and hotel reviews, we observe that flat or slightly increasing affinity scores translate to bowl-shaped ratio scores and normalized bias scores (Fig. 5A). In other words, shopping trips where a large majority of purchases are from one product category are much more common than expected by chance. This matches the intuition that many shopping trips can be categorized as grocery runs or clothes shopping trips. Our results also highlight an intuitive difference between these types of trips: It is more common to pick up a small number of grocery items while on a clothes shopping trip than to purchase a small number of clothes items while on a grocery run. This is reflected in the larger gaps between affinity scores for groceries and can also been seen in the normalized bias scores. In particular, the type-*k* normalized bias score **f***_k_* for groceries is very close to 1 for all values of *k*, indicating that simple homophily for groceries is almost as high as it possibly could be. Meanwhile, normalized biased scores **f***_t_* are very close to −1 if *t* is just a few values less than *k* (e.g., when *t* = 9 for the *k* = 12 plot), indicating that grocery trips that include a few clothing items are almost as far below baseline scores as they could be.

### Gender homophily in pictures

**Fig. 6.**
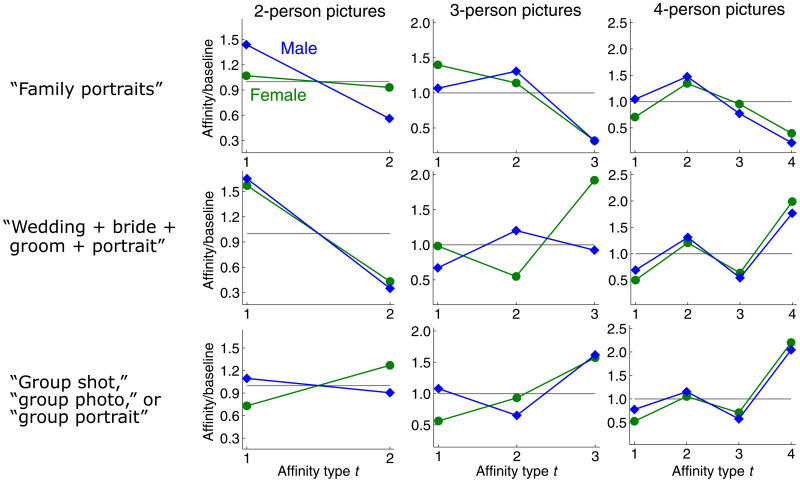
Ratio scores with respect to gender for three collections of group pictures, obtained via three image search queries on Flickr. Normalized bias scores capture the same trends. Hypergraph measures provide richer information than graph homophily indices obtained by collapsing pictures into pairwise relationships based on coappearance. For family pictures (top row), ratio scores capture the intuition that all-male and all-female family pictures are statistically uncommon, as shown by low ratio scores for three- and four-person pictures of all men or all women. Meanwhile, the graph homophily indices for men and women when collapsing all family pictures into pairwise relationships are 0.43 and 0.41, respectively, just below the baseline of 0.5 for balanced classes. Ratio scores for four-person wedding pictures (middle row) or general group pictures (bottom row) indicate a high frequency of social gatherings of all men or all women. The slightly higher-than-random affinities for gatherings with two men and two women are possibly due to a high number of pictures of two opposite-gender couples. Two-person wedding photos are likely to be of a bride and groom, which is reflected in low type-2 ratio scores (first column, middle row). However, pictures with three or four people are often gender homogeneous. This information is lost when collapsing all pictures into pairwise coappearances, in which case the resulting graph homophily indices of 0.57 and 0.55 are both slightly above baseline (0.5). Results for the dataset are robust to perturbations; we see similar patterns from average affinity scores obtained from different subsamples of each dataset (see the Supplementary Materials).

Our framework can also be used to study homophily in groups even when group members are not uniquely associated with nodes in a hypergraph. We apply our framework to analyze gender homophily in group pictures, composed of three subsets of pictures capturing family portraits, wedding portraits, and general group pictures (*29*). To compute affinity scores, it suffices to know the size and gender composition of each group in a picture, even without unique identifiers for each individual. Hypergraph affinity scores reveal that the gender distribution in group pictures depends on group size. This information is completely lost if we reduce group pictures to pairwise coappearances and compute a graph homophily index. Our framework also reveals several salient differences between gender distributions across different picture types (Fig. 6A).

In wedding pictures, affinity scores for same-gender two-person pictures are far smaller than expected at random, reflecting the fact that many of these photos are of a bride and groom. However, there is more gender homophily in three- and four-person pictures at weddings, as shown by higher-than-random affinity scores for gender homogeneous groups (i.e., simple homophily). Reducing all wedding pictures to pairwise relationships suppresses these subtle differences and just produces a graph where both genders have slightly higher-than-random graph homophily indices. Wedding pictures and general group pictures with exactly four people show similar patterns. Pictures with two men and two women are slightly more common than expected by chance, and pictures of all men or all women are much more common than expected by chance. The former may be due to a high volume of pictures of two heterosexual couples, while the latter indicates an overall tendency for friends to gather in groups that are completely homogeneous with respect to gender.

Our framework reveals several interesting differences in the context of family pictures. In family pictures with three or four people, pictures with only men or only women are far less common than expected by chance. This matches the intuition that statistically, family photos are less likely to be of all men or all women. In contrast, graph homophily indices for men and women on a reduced graph (defined by coappearances) are only slightly lower than expected by chance. Another interesting observation is that for four-person pictures, type-2 ratio scores are the highest for both men and women. This can be explained by the fact that it is statistically very common for four-person families to consist of a mother and father with two children. In this case, assuming that the children have an equally likely chance of being male or female, there is a 50% chance that there will be one boy and one girl, a 25% chance that both children will be boys, and a 25% chance that both children will be girls. This explains why type-2 ratio scores are much higher than type-3 ratio scores. Again, this type of nuanced information is lost when reducing group pictures to coappearances and using graph measures of homophily.

## DISCUSSION

Understanding group formation and interactions has long been a goal of homophily research, but previous methods have focused almost exclusively on pairwise approaches. Our framework for hypergraph homophily quantifies the tendency of individuals to participate in multiway interactions that differ in size and class balance. Our results show that group interactions among different classes of individuals must obey certain combinatorial constraints, which render seemingly intuitive notions of group homophily impossible. At the same time, these combinatorial impossibilities do not imply that group interactions happen indiscriminately of class labels, and in practice, we do see many examples of class homogeneity in group interactions. We find that in many group settings, homophily can be characterized by bowl-shaped ratio score curves. These scores indicate that different classes of individuals exhibit increasing and higher-than-random affinities for group interactions when a large enough majority of group members are from the same class.

Our empirical results illustrate the utility of defining and computing a different affinity score for each group size and group type separately. This is most clearly illustrated in our results on group pictures, where homophily patterns are significantly affected both by picture context (e.g., wedding versus family picture) and group size. At the same time, it can also be useful to capture aggregate information about group homophily that persists across group types and sizes. Our measures of MoHI and MaHI provide one simplified aggregate score; determining other aggregate scores that summarize the tendency toward homophily across multiple group types and sizes at once is an interesting direction for future research. Another direction to consider is how our framework can be applied and generalized to hypergraphs with multiple class labels. Our definition of affinity and baseline scores can already be applied to an arbitrary number of class labels, but an interesting open question to explore is how combinatorial limits change in this setting. Finally, it would be worthwhile to consider how our hypergraph framework can be used to explore higher-order generalizations of other mixing patterns, such as monophily (*16*), that may be present in group interactions even when homophily is not.

## MATERIALS AND METHODS

The Supplementary Materials provides details for the original datasets and the construction of each hypergraph from Figs. 2 to 6.

### Asymptotic baseline scores

For numerical experiments on all hypergraphs except for the small hypergraph used in Fig. 5, we used asymptotic variant of the standard baseline scores in Eq. 2. To compute asymptotic baselines, we consider a two-class hypergraph where the class proportions are given by $\alpha =\frac{|A|}{n}$ and $(1-\alpha )=\frac{|B|}{n}$.

Treating α as a constant and letting *n* → ∞, the standard baseline scores converge to the following asymptotic baselines

$$b_{t}(A)=\alpha^{t-1}{\left(1-\alpha \right)}^{k-t}\left(\begin{array}{c} k-1\\ t-1 \end{array}\right)$$ (16)

$$b_{t}(B)={\left(1-\alpha \right)}^{t-1}\alpha^{k-t}\left(\begin{array}{c} k-1\\ t-1 \end{array}\right)$$ (17)

for *t* ∈ [*k*]. These scores correspond to probability mass functions for binomial random variables Bin(α, *k*) and Bin(1 − α, *k*), respectively. The number of nodes *n* is sufficiently large for our datasets that these are virtually indistinguishable from standard baseline scores and are also more convenient to compute and use in practice. For standard baselines scores, computing binomial coefficients for large *n* and *k* can lead to overflow issues in practice; asymptotic baselines provide one way to avoid this issue. This also mirrors the standard practice in the graph case, since typically the graph homophily index for a class *X* is compared against the asymptotic baseline score ∣*X* ∣/*n* rather than ( ∣*X* ∣ − 1)/(*n* − 1).

### Graph homophily index

In some cases, we compare against the graph homophily index obtained by reducing hyperedges in the hypergraph *H* = (*V*, *E*) to pairwise relationships. Formally, we define a graph *G* where nodes *u*, *v* ∈ *V* share an edge if {*u*, *v*} ⊆ *e* for some *e* ∈ *E*. The graph *G* can be described as a two-uniform hypergraph, and the graph homophily index for a class *X* ⊆ *V* is exactly the type-2 affinity **h**_2_(*X*), computed using Eq. 1.

## Supplementary Materials

### This PDF file includes:

Supplementary Text Table S1 Figs. S1 to S4 References
