# Supplementary material for: Combinatorial characterizations and impossibilities for higher-order homophily
Source: Sci Adv. 2023 Jan 6;9(1):eabq3200. doi: 10.1126/sciadv.abq3200 (PMC9821936; doi:10.1126/sciadv.abq3200)
Supplement: Supplementary file 1 — Supplementary Text Table S1 Figs. S1 to S4 References [file sciadv.abq3200_sm.pdf]

Science Advances
AAAS

Supplementary Materials for

Combinatorial characterizations and impossibilities for higher-order homophily

Nate Veldt *et al.*

Corresponding author: Nate Veldt, nveldt@tamu.edu

Sci. Adv. **9**, eabq3200 (2023)
DOI: 10.1126/sciadv.abq3200

This PDF file includes:

Supplementary Text
Table S1
Figs. S1 to S4
References

Supplementary Material for

Combinatorial Characterizations and Impossibilities for Higher-order Homophily

Nate Veldt,* Austin R. Benson, Jon Kleinberg
*Corresponding author. E-mail: nveldt@tamu.edu

This PDF Includes

Supplementary Text
Table S1
Supplementary Figures S1 to S4

Supplementary Text

1 Main Theoretical Results for Hypergraph Affinities

In this section we provide full details for our theoretical results regarding hypergraph affinity scores. We begin by reviewing and covering additional necessary terminology and notation in Section 1.1. We then show how to interpret affinity scores as maximum likelihood estimates for a certain affinity parameter of a binomial model for degree data (Section 1.3), cover additional background on baseline scores (Section 1.4), and then prove our main theoretical impossibility results for hypergraph homophily (Section 2). In Section 3, we show how these results can be adapted to an alternative notion of affinity scores.

1.1 Notation and Terminology

Consider a hypergraph $H = (V, E)$ where V is a set of $n = |V|$ nodes and E is a set of $m = |E|$ hyperedges. We assume throughout that H is k-uniform (where k is constant) and non-degenerate, meaning that all hyperedges are of a fixed size k, and a node can appear at most once in a hyperedge. We also assume that nodes are organized into one of two classes $A \subseteq V$ and $B \subseteq V$ where $A \cup B = V$ and $A \cap B = \emptyset$.

For class $X \in \{A, B\}$ and integer $t \in [k] = \{1, 2, \dots k\}$, we say that a hyperedge $e \in E$ is of type-(X, t) if exactly t nodes in e are from class X. Let $m_t(X)$ denote the number of type-(X, t) hyperedges in E. Since there are exactly two node classes, $m_t(A) = m_{k-t}(B)$ and $m_t(B) = m_{k-t}(A)$. It is often convenient to refer to hyperedge types in an absolute sense, without specifying class. We say that a hyperedge is of absolute type-t if exactly t of its nodes are from class A, and denote the number of such edges by

$$m_t = m_t(A) = \text{number of hyperedges of absolute type-}t \text{ in } E. \quad (1)$$

The degree of a node $v \in V$, denoted $d(v)$, is the number of hyperedges it participates in. For an integer $t \in [k]$, let $d_t(v)$ denote the number of hyperedges v participates in where exactly t nodes are from v's class,

including v itself. We refer to this as the type-t degree of v. Summing typed-degrees produces the degree of a node:

$$d(v) = \sum_{t=1}^{k} d_t(v).$$

The type-t affinity score for a class $X \in \{A, B\}$ measures the propensity for nodes in this class to participate in type-(X, t) hyperedges. This score can be expressed in terms of sums of node degrees:

$$h_t(X) = \frac{\sum_{v \in X} d_t(v)}{\sum_{v \in X} d(v)}. \quad (2)$$

This directly generalizes the homophily index of a graph [16], which is defined similarly in terms of typed degrees, and corresponds to the case where $k = t = 2$.

Affinity scores can also be expressed in terms of hyperedge counts. The sum of type-t degrees for a class X satisfies

$$\sum_{v \in X} d_t(v) = t m_t(X)$$

The value $m_t(X)$ is scaled by a factor t to account for the fact that each type-(X, t) hyperedge affects the degree of t different nodes from class X. We can express type-t affinity scores for both classes in terms of absolute hyperedge types as follows:

$$\mathbf{h}_t(A) = \frac{t m_t}{\sum_{i=1}^{k} i m_i} \quad (3)$$

$$\mathbf{h}_t(B) = \frac{t m_{k-t}}{\sum_{i=1}^{k} i m_{k-i}}. \quad (4)$$

1.2 Cardinality-Based Hypergraph Stochastic Block Model

In order to provide a statistical interpretation for hypergraph affinity scores, we define a simple new generative model for hypergraphs. For this model, consider a set of nodes V separated into two classes A and B. We say a tuple of k distinct nodes in V is a type-t k-tuple if exactly t of nodes in the tuple are from class A. For each $t \in \{0, 1, \ldots, k\}$, define a probability $p_t \in [0, 1]$. We emphasize the fact that these probabilities are defined with respect to a fixed class A; since it is possible to have hyperedges where all nodes are from class B, this also includes a probability p_0. We define the *cardinality-based hypergraph stochastic block model* (cardinality-based HSBM) as follows: for each type-t k-tuple of nodes $T = (v_1, v_2, \ldots, v_k)$, we generate a hyperedge on T with probability p_t. We denote the distribution of cardinality-based hypergraph stochastic block models with size k hyperedges by $\mathcal{H}(n, k, A, B, \mathbf{p})$ where $\mathbf{p} = [p_0, p_1, \ldots, p_k]$ is a vector of hyperedge probabilities. This a special case of the general k-uniform hypergraph stochastic block model [30], which may involve more than two ground truth clusters or classes.

1.3 Affinity Scores as Maximum Likelihood Estimates

We now show how affinity scores for class A can be derived as maximum likelihood estimates for an affinity parameter of a certain binomial distribution. The same approach could also be applied to class B.

1.3.1 Type-degree Random Variables

Assume we are given a k-uniform hypergraph from the cardinality-based HSBM $\mathcal{H}(n, k, A, B, \mathbf{p})$, where the probability vector $\mathbf{p} = [p_0, p_1, \ldots, p_k]$ is given up front and fixed. For a node $a \in A$, let T_j be the total

number of type-j k-tuples of nodes that a belongs to:

$$T_j = \binom{|A|-1}{j-1}\binom{|B|}{k-j}. \tag{5}$$

This value is the same regardless of which $a \in A$ we consider, and represents the maximum number of type-j hyperedges that a could belong to in an n-node hypergraph with node classes A and B. The type-t degree of each node $a \in A$ conditioned on probability p_t will be a binomial random variable

$$D_t(a) \sim \text{Binom}\left(T_t, p_t\right). \tag{6}$$

We also define a random variable $D(a)$ for the total degree of $a \in A$ by

$$D(a) = \sum_{j=1}^{k} D_j(a). \tag{7}$$

Finally, define another random variable for measuring the contribution to $D(a)$ made by hyperedges that are *not* of type-t:

$$\tilde{D}_t(a) = \sum_{j=1, j\neq t}^{k} D_j(a). \tag{8}$$

For any fixed t, $D_t(a) + \tilde{D}_t(a) = D(a)$. The degree random variables $D(a)$ will not be independent for different $a \in V$, since the degrees must define a valid degree sequence for a k-uniform hypergraph. However, we can prove that they will be approximately independent by adapting existing techniques for graphs [31].

Supplemental Lemma 1. *Let $H \sim \mathcal{H}(n, k, A, B, \boldsymbol{p})$ be a cardinality-based HSBM with hyperedge parameters satisfying $\hat{p} = \max_i p_i = O\left(\frac{1}{n^{k-1}}\right)$. If $\ell > k$ is a fixed constant, the degree random variables for any set of ℓ nodes, $D(1), D(2), \ldots, D(\ell)$, are asymptotically independent.*

Proof. Let $\mathcal{K}$ denote the set of k-tuples of nodes in H, and let L denote the set of ℓ nodes we are considering, which we denote by $\{1, 2, \ldots, \ell\}$ without loss of generality. Each $e \in \mathcal{K}$ is associated with a Bernoulli random variable X_e such that $X_e = 1$ if an edge is placed at k-tuple e. Each random variable $D(i)$ for $i \in [\ell]$ is a sum of Bernoulli random variables:

$$D(i) = \sum_{e\colon i \in e} X_e.$$

For $i, j \in [\ell]$, $D(i)$ and $D(j)$ are not independent, as X_e appears in both of their sums whenever $i \in e$ and $j \in e$. If e is a k-tuple of nodes from L, then X_e contributes to the sum of all of the random variables $(D_i)_{i\in L}$. In general for an arbitrary $e \in \mathcal{K}$, the variable X_e shows up in $|e \cap L|$ of these degree variables. Note that for each $s \in \{2, 3, \ldots k\}$, there are $\binom{\ell}{s}\binom{n-\ell}{k-s}$ distinct k-tuples involving s nodes from L and $k - s$ nodes from $V - L$.

In order to prove that random variables $(D_i)_{i\in L}$ are approximately independent, for each $i \in L$ we construct a new random variable $\hat{D}(i)$ in such a way that $D(i)$ and $\hat{D}(i)$ have the same distribution, and so that the $\hat{D}(i)$ variables are mutually independent. In order to establish asymptotic independence of the original $D(i)$ variables, we then prove that

$$\text{Pr}\left[(D(i))_{i\in L} \neq (\hat{D}(i))_{i\in L}\right] = o(1).$$

In order to accomplish this, for each X_e that shows up in more than one variable from $(D_i)_{i\in L}$, we construct $|e \cap L| - 1$ other *independent* copies of this random variable X_e, denoted by $X_e^{(2)}, X_e^{(3)}, \ldots, X_e^{(s)}$ where $s = |e \cap L|$. Define $X_e^{(1)} = X_e$ for notational convenience. We then define a new variable $\hat{D}(i)$ for each $i \in L$, which is the same as $D(i)$, except we carefully replace the X_e variables with the independent copies of X_e, in order to ensure the $\hat{D}(i)$ variables are independent. We begin by defining

$$\hat{D}(1) = \sum_{e\colon 1\in e} X_e^{(1)} = D(1).$$

Then, to define $\hat{D}(j)$ for $j > 1$, we start with the same sum of random variables that defines $D(j)$, but then we replace each X_e in this sum by the copy $X_e^{(r+1)}$, where r is the number of times that X_e appeared in a sum $D(i)$ for $i < j$. Thus, if X_e shows up s times in the summations defining $(D_i)_{i\in L}$, we have constructed s independent copies of X_e and used these in defining $(\hat{D}_i)_{i\in L}$. As a result, $\hat{D}(i)$ and $D(i)$ have the same distribution, but the variables $(\hat{D}_i)_{i\in L}$ are independent.

What remains is to prove that the probability that $(\hat{D}_i)_{i\in L}$ and $(D_i)_{i\in L}$ are not equal goes to zero. These random variables will be the same if for every k-tuple e with $s = |e \cap L| \geq 2$, the variables $X_e^{(1)}, \ldots, X_e^{(s)}$ are all the same. Each of these is a Bernoulli random variable with some probability $p_t \leq \hat{p} = O\left(\frac{1}{n^{k-1}}\right)$. The probability that $X_e^{(1)}, \ldots, X_e^{(s)}$ all coincide is the probability that they all equal one or all equal zero, so:

$$\Pr(X_e^{(1)}, \ldots, X_e^{(s)} \text{ do not coincide}) = 1 - p_t^s - (1-p_t)^s \leq 1 - (1-\hat{p})^s. \tag{9}$$

For $s \in \{2, 3, \ldots, k\}$, let $\mathcal{K}_s$ denote the set of k-tuples in with exactly s nodes from L. We can use Boole's inequality to bound the probability that $(\hat{D}_i)_{i\in L}$ and $(D_i)_{i\in L}$ are not equal:

$$\begin{aligned}
\Pr\left[(\hat{D}_i)_{i\in L} \neq (D_i)_{i\in L}\right] &\leq \sum_{s=2}^{k} \sum_{e\in\mathcal{K}_s} \Pr(X_e^{(1)}, \ldots, X_e^{(s)} \text{ do not coincide}) \\
&\leq \sum_{s=2}^{k} \sum_{e\in\mathcal{K}_s} 1 - (1-\hat{p})^s \\
&\leq \sum_{s=2}^{k} \binom{\ell}{s}\binom{n-\ell}{k-s}\left(1 - (1-\hat{p})^s\right).
\end{aligned}$$

Finally, if $\hat{p} = O(n^{-\alpha})$ for some integer α, we know that $\hat{p} \leq \frac{C}{n^\alpha}$ for some constant C, so we have the following asymptotic result:

$$\begin{aligned}
\binom{n-\ell}{k-s}(1-(1-\hat{p})^s) &\leq \binom{n-\ell}{k-s}\left(1 - \left(1 - \frac{C}{n^\alpha}\right)^s\right) \\
&= \binom{n-\ell}{k-s}\left(\frac{n^{\alpha s} - (n^\alpha - C)^s}{n^{\alpha s}}\right) \\
&= O\left(\frac{n^{k-s}\cdot n^{\alpha(s-1)}}{n^{\alpha s}}\right) \\
&= O\left(\frac{n^{k-s}}{n^\alpha}\right).
\end{aligned}$$

where we have used the fact that $(n^\alpha - C)^s = n^{\alpha s} - Cn^{\alpha(s-1)} + f(n)$ where $f(n)$ represents lower order terms in n. Thus, as long as $\alpha \geq k-1 > k-s$, we see that this entire expression goes to zero for every

$s \in \{2, 3, \ldots, k\}$, and so we have our desired asymptotic result:

$$\lim_{n\to\infty} \Pr\left[(\hat{D}_i)_{i\in L} \neq (D_i)_{i\in L}\right] \leq \lim_{n\to\infty} \sum_{s=2}^{k} \binom{\ell}{s}\binom{n-\ell}{k-s}(1-(1-\hat{p})^s) = 0.$$

□

1.3.2 Conditional Distribution of Type-t Degrees

Assume we observe a two-class k-uniform hypergraph for which the degree of node $a \in A$ is given by $d(a)$. Given our fixed hyperedge-probabilities p_j for each edge type, we can prove that for a fixed t, the conditional random variable $(D_t(a) \mid D_a = d(a))$ is asymptotically binomially distributed

$$D_t(a) \mid d(a) \sim \text{Binom}\left(d(a), f_t\right), \tag{10}$$

where f_t is the affinity parameter:

$$f_t = \frac{p_t \cdot T_t}{\displaystyle\sum_{j=1}^{k} p_j \cdot T_j}. \tag{11}$$

This holds specifically for the parameter regime where T_j is large but $p_j \cdot T_j$ is constant for all $j \in \{1, 1, \ldots k\}$, and each $d(a)$ is a constant. Recall that $D_j(a)$ is binomially distributed for all $j \in \{1, \ldots, k\}$. Under the assumed conditions on p_j and T_j, these binomial distributions are asymptotically equivalent to Poisson distributions:

$$\lim_{n\to\infty} \Pr(D_j(a) = c) = \frac{(p_j \cdot T_j)^c}{c!} e^{-p_j \cdot T_j}. \tag{12}$$

A sum of binomials is also asymptotically equivalent to a Poisson distribution with the same mean [32], so we also have that

$$\lim_{n\to\infty} \Pr\left(\sum_{j=1}^{k} D_j(a) = c\right) = \frac{(\sum_{j=1}^{k} p_j \cdot T_j)^c}{c!} e^{-(\sum_{j=1}^{k} p_j \cdot T_j)}. \tag{13}$$

Similarly,

$$\lim_{n\to\infty} \Pr\left(\sum_{j=1}^{k} \tilde{D}_j(a) = d(a) - c\right) = \frac{(\sum_{j\neq t} p_j \cdot T_j)^{d(a)-c}}{(d(a)-c)!} e^{-\sum_{j\neq t} p_j \cdot T_j} \tag{14}$$

By our assumption that $p_j T_j = O(1)$, the right hand sides of (12), (13), and (14) are all constants. Therefore, asymptotically the distribution of $(D_t(a) | D_a = d(a))$ is a binomial with parameter f_t, since

$$\begin{aligned}
\Pr(D_t(a) = c | D_a = d(a)) &= \Pr\left(D_t(a) = c \,\middle|\, \sum_{j=1}^{k} D_j(a) = d(a)\right) \\
&= \frac{\Pr(D_t(a) = c, \tilde{D}_t(a) = d(a) - c)}{\Pr(\sum_{j=1}^{k} D_j(a) = d(a))} \\
&= \frac{\Pr(D_t(a) = c) \cdot \Pr(\tilde{D}_t(a) = d(a) - c)}{\Pr(\sum_{j=1}^{k} D_j(a) = d(a))}
\end{aligned}$$

and therefore

$$\lim_{n\to\infty} \Pr(D_t(a) = c \mid D_a = d(a)) = \frac{\left[\frac{(p_t \cdot T_t)^c}{c!} e^{-p_t \cdot T_t}\right] \left[\frac{(\sum_{j\neq t} p_j \cdot T_j)^{d(a)-c}}{(d(a)-c)!} e^{-\sum_{j\neq t} p_j \cdot T_j}\right]}{\left[\frac{(\sum_j p_j \cdot T_j)^{d(a)}}{(d(a))!} e^{-\sum_j p_j \cdot T_j}\right]}$$

$$= \binom{d(a)}{c} \left(\frac{p_t \cdot T_t}{\sum_j p_j \cdot T_j}\right)^c \quad 1 - \frac{p_t \cdot T_t}{\sum_j p_j \cdot T_j}\Bigg)^{d(a)-c}.$$

1.3.3 Affinity Index as Maximum Likelihood Estimate

Given observed degree data $(d(a), d_t(a) | a \in A)$ for a two-class, k-uniform hypergraph, we can model the type-t degree for an arbitrary node in A using the binomial distribution given in (10). For this data, the type-t affinity score will equal the maximum likelihood estimate for the affinity parameter f_t. To show this result, define $\tilde{d}_t(a) = d(a) - d_t(a)$, i.e., the part of the degree that does not come from type-t hyperedges. For simplicity and without loss of generality, denote the nodes in A by the indices $1, 2, \ldots, |A|$. Treating degrees as independent, the likelihood function for observing the given degrees for a given parameter f_t is

$$L(f_t) = \Pr(D_t(1) = d_t(1), D_t(2) = d_t(2), \ldots, D_t(|A|) = d_t(|A|))$$
$$= \prod_{a\in A} \binom{d(a)}{d_t(a)} \cdot {f_t}^{d_t(a)} \cdot (1 - f_t)^{\tilde{d}_t(a)}.$$

The log-likelihood function is

$$\ell(f_t) = \log L(f_t) = \sum_{a\in A} \log \binom{d(a)}{d_t(a)} + \log({f_t}^{d_t(a)}) + \log((1 - f_t)^{\tilde{d}_t(a)})$$
$$\propto \sum_{a\in A} d_t(a) \log(f_t) + \tilde{d}_t(a) \log(1 - f_t).$$

Taking the derivative with respect to the parameter f_t and setting it equal to zero, we find that the log-likelihood is maximized then f_t is exactly equal to the type-t affinity index.

$$\frac{\partial \ell}{\partial f_t} = 0 \Rightarrow \frac{\sum_{a\in A} d_t(a)}{f_t} = \frac{\sum_{a\in A} \tilde{d}_t(a)}{1 - f_t} \Rightarrow f_t = \frac{\sum_{a\in A} d_t(a)}{\sum_{a\in A} d(a)}.$$

1.4 Baseline Scores and Proofs of Propositions 1 and 2

In order to determine the meaningfulness of a type-t affinity score, we compare it against a baseline score representing a null probability for participation in type-t hyperedges. For class X, the standard type-t baseline score $\mathbf{b}_t(X)$ measures the probability that a node $v \in X$ forms a type-(X, t) hyperedge (i.e., there are exactly t nodes from v's class in the hyperedge) if it selects $k - 1$ other nodes from V uniformly at random. Formally,

$$\mathbf{b}_t(X) = \frac{\binom{|X|-1}{t-1}\binom{n-|X|}{k-t}}{\binom{n-1}{k-1}}. \tag{15}$$

Comparing the type-t affinity $\mathbf{h}_t(X)$ against $\mathbf{b}_t(X)$ generalizes a standard approach for checking for homophily in graphs. When $k = t = 2$, the hypergraph affinity score $\mathbf{h}_t(X)$ equals the homophily index of a graph, and the type-2 baseline score is

$$\mathbf{b}_2(X) = \frac{|X| - 1}{n - 1}. \tag{16}$$

As $n \to \infty$, this converges to the class proportion $|X|/n$, which is typically used as the standard baseline for the homophily index of a graph. In addition to generalizing the baseline for a graph homophily index, our hypergraph baseline scores satisfy the following intuitive interpretation, as given in the main text.

Proposition 1. *Let $H^*_{k,n} = (V, E)$ be the complete k-uniform hypergraph on n nodes. The type-t affinity score for class $X \subseteq V$ equal the type-t baseline score in (15).*

Proof. The number of type-(X, t) hyperedges in H is $m_t(X) = \binom{|X|}{t}\binom{n-|X|}{k-t}$, the total number of ways to choose t nodes from X and $k - t$ nodes that are not in X. The type-t affinity score for class X is therefore

$$\mathbf{h}_t(X) = \frac{t m_t(X)}{\sum_{i=1}^k i m_i(X)} = \frac{t\binom{|X|}{t}\binom{n-|X|}{k-t}}{\sum_{i=1}^k i\binom{|X|}{i}\binom{n-|X|}{k-i}}$$

$$= \frac{|X|\binom{|X|-1}{t-1}\binom{n-|X|}{k-t}}{|X|\sum_{i=1}^k \binom{|X|-1}{i-1}\binom{n-|X|}{k-i}} = \frac{\binom{|X|-1}{t-1}\binom{n-|X|}{k-t}}{\sum_{i=1}^k \binom{|X|-1}{i-1}\binom{n-|X|}{k-i}},$$

where we have used the fact that $\binom{|X|}{i} = \frac{|X|}{i}\binom{|X|-1}{i-1}$. This is the same as $\mathbf{b}_t(X)$ — the numerator is identical, and the denominator is an alternative way to list all the possible ways to select $k-1$ nodes from a set of $n-1$ nodes, by separately counting how many of each type of hyperedge could be formed. □

To provide further intuition for the baseline scores, we observe that the type-t baseline score is also related the type-t hypergraph affinity for a hypergraph obtained by generating hyperedges at random without regard to node class. Consider a cardinality-based HSBM where for some $p \in (0, 1)$, $p_i = p$ for all $i \in \{0, 1, 2, \dots, k\}$. If M_t is a random variable representing the number of type-t hyperedges, then

$$\mathbb{E}[M_t] = p \cdot \binom{|A|}{t}\binom{n-|A|}{k-t}$$

If we replace m_t with this expected value $\mathbb{E}[M_t]$ in the definition for $\mathbf{h}_t(A)$ given in equation (3), then we exactly recover the type-t baseline score for class A:

$$\frac{t\mathbb{E}[M_t]}{\sum_{i=1}^k i \cdot \mathbb{E}[M_i]} = \frac{p \cdot t \cdot \binom{|A|}{t}\binom{n-|A|}{k-t}}{p \cdot \sum_{i=1}^k i \cdot \binom{|A|}{i}\binom{n-|A|}{k-i}} = \frac{|A|\binom{|A|-1}{t-1}\binom{n-|A|}{k-t}}{|A|\sum_{i=1}^k \binom{|A|-1}{i-1}\binom{n-|A|}{k-i}} = \mathbf{b}_t(A). \tag{17}$$

An analogous result also holds for baselines scores of class B.

Proposition 2 from the main text is an even stronger result, showing that when hyperedges are generated at random without regard for node labels, the ratio scores of the resulting hypergraph converge to one.

Proposition 2. *Fix any $p \in (0, 1)$ and a positive integer k, and let $H = (V, E)$ be a random hypergraph on n nodes that is formed by turning each k-tuple of nodes in V into a hyperedge with probability p. As $n \to \infty$, the ratio scores for a class $X \subseteq V$ with $|X| = \Theta(n)$ converge in probability to 1.*

Proof. Our goal is to show that for every $\varepsilon > 0$ and $\delta > 0$, there exists some $n_0 \in \mathbb{N}$ such that for all $n > n_0$ and for all $t \in [k]$, with probability at least $1 - \delta$,

$$\left| \frac{\mathbf{h}_t(X)}{\mathbf{b}_t(X)} - 1 \right| < \varepsilon.$$

For $i \in [k]$, let N_i be the number of k-tuples with exactly i nodes from class X, and M_i be the expected number of hyperedges of type-(X, i) (exactly i nodes from class X) in H. The random variable M_i is binomially distributed, $M_i \sim \text{Bin}(p, N_i)$, and has the following expected value and variance:

$$\begin{aligned} m_i &= \mathbb{E}[M_i] = pN_i \\ \sigma_i^2 &= N_i p(1-p). \end{aligned}$$

As long as $M_i > 0$ for some $i \in [k]$, the type-t affinity score for H is well defined and equals

$$\mathbf{h}_t(X) = \frac{tM_t}{\sum_{i=1}^k iM_i}. \tag{18}$$

From (17) we know that replacing M_i with m_i in (18) yields the type-t baseline score for H:

$$\mathbf{b}_t(X) = \frac{tm_t}{\sum_{i=1}^k im_i}.$$

To prove that $\mathbf{h}_t(X)/\mathbf{b}_t(X)$ converges to one, we first prove several facts about the limiting behavior of M_i/m_i. By Chebyshev's inequality, we know that for any $i \in [k]$ and any $\varepsilon > 0$,

$$\Pr\left(\left| \frac{M_i}{m_i} - 1 \right| \geq \varepsilon \right) \leq \frac{1}{N_i p(1-p)\varepsilon^2}. \tag{19}$$

Since $N_i = O(n^k)$ for every $i \in [k]$, this establishes that as long as n is large enough, M_i/m_i can be made arbitrarily close to one with high probability. With this in mind, fix $\varepsilon > 0$ and $\delta > 0$, and choose n_0 so that for all $n > n_0$, with probability at least $1 - \delta$

$$\left| \frac{M_i}{m_i} - 1 \right| < \hat{\varepsilon}, \tag{20}$$

for all $i \in [k]$, where $\hat{\varepsilon} < \min\left\{\frac{1}{2}, \frac{\varepsilon}{4}\right.$. This implies that $M_i > 0$ and $(1 - \hat{\varepsilon}) < \frac{M_i}{m_i} < (1 + \hat{\varepsilon})$, and therefore we also know that

$$\left| \frac{m_i}{M_i} - 1 \right| = \frac{m_i}{M_i} \left| \frac{M_i}{m_i} - 1 \right| < \frac{\hat{\varepsilon}}{1 - \hat{\varepsilon}}. \tag{21}$$

For our final step of the proof we will make use of the following useful inequality, that holds for two sets of positive numbers $\{a_1, a_2, \ldots, a_\ell\}$ and $\{b_1, b_2, \ldots, b_\ell\}$ and an arbitrary integer ℓ:

$$\frac{\sum_{i=1}^\ell a_i}{\sum_{i=1}^\ell b_i} \leq \max_{i \in [\ell]} \frac{a_i}{b_i}. \tag{22}$$

Using (20), (21), and (22), we know that with probability at least $1-\delta$,

$$
\begin{aligned}
\left|\frac{\mathbf{h}_t(A)}{\mathbf{b}_t(A)}-1\right| &= \left|\frac{tM_t}{\sum_{i=1}^k iM_i}\frac{\sum_{i=1}^k im_i}{tm_t}-1\right| = \left|\frac{(M_t\sum_{i=1}^k im_i)-(m_t\sum_{i=1}^k iM_i)}{m_t\sum_{i=1}^k iM_i}\right| \\
&= \left|\frac{(M_t\sum_{i=1}^k im_i)-(m_t\sum_{i=1}^k im_i)+(m_t\sum_{i=1}^k im_i)-(m_t\sum_{i=1}^k iM_i)}{m_t\sum_{i=1}^k iM_i}\right| \\
&\leq \left|\frac{(M_t\sum_{i=1}^k im_i)-(m_t\sum_{i=1}^k im_i)}{m_t\sum_{i=1}^k iM_i}\right| + \left|\frac{(m_t\sum_{i=1}^k im_i)-(m_t\sum_{i=1}^k iM_i)}{m_t\sum_{i=1}^k iM_i}\right| \\
&\leq \frac{|M_t-m_t|}{m_t}\frac{\sum_{i=1}^k im_i}{\sum_{i=1}^k iM_i}+\frac{\sum_{i=1}^k i|m_i-M_i|}{\sum_{i=1}^k iM_i} \\
&\leq \left|\frac{M_t}{m_t}-1\right|\max_i\frac{m_i}{M_i}+\max_i\frac{|m_i-M_i|}{M_i} \qquad \text{(applying (22))} \\
&= \left|\frac{M_t}{m_t}-1\right|\frac{m_j}{M_j}+\left|\frac{m_\ell}{M_\ell}-1\right| \qquad \text{(for some integers } j \text{ and } \ell) \\
&< \frac{\hat{\varepsilon}}{1-\hat{\varepsilon}}+\frac{\hat{\varepsilon}}{1-\hat{\varepsilon}} < 4\hat{\varepsilon} < \varepsilon.
\end{aligned}
$$

□

2 Hypergraph Homophily Impossibility Results

We restate our main results for hypergraph homophily, exactly as given in the main text.

Theorem 3. *Let H be a two-class, k-uniform hypergraph and $\{\boldsymbol{b}_i(X)\colon i\in[k], X\in\{A,B\}\}$ be realizable baseline scores. For odd k, it is impossible for both classes to exhibit monotonic homophily.*

Theorem 4. *Let H be a two-class, k-uniform hypergraph and $\{\boldsymbol{b}_i(X)\colon i\in[k], X\in\{A,B\}\}$ be realizable baseline scores. If k is even, then it is imposssible for both classes satisfy monotonic homophily if additionally $\frac{\boldsymbol{h}_\ell(X)}{\boldsymbol{b}_\ell(X)} > \frac{\boldsymbol{h}_{\ell-1}(X)}{\boldsymbol{b}_{\ell-1}(X)}$ for one class $X\in\{A,B\}$, where $\ell=k/2$.*

Theorem 5. *Let $H=(V,E)$ be a two-class k-uniform hypergraph and $\{\boldsymbol{b}_i(X)\colon i\in[k], X\in\{A,B\}\}$ be realizable baseline scores.*

- *If k is odd, it is impossible for both classes to simultaneously exhibit majority homophily*
- *If k is even, it is impossible for both classes to exhibit majority homophily if additionally $\boldsymbol{h}_{k/2}(X) > \boldsymbol{b}_{k/2}(X)$ for one of the classes $X\in\{A,B\}$.*

In the main text we include a full proof of Theorem 3. Theorem 4 is nearly identical and relies on simply adding one extra constraint and repeating the same basic steps. Here we provide full details for our majority homophily result for odd k, and show how they can be altered to yield the result for even k. For clarity and ease of presentation, we include many of the same steps as given in the main text, in some cases with expanded explanations.

Throughout the section, $H=(V,E)$ denotes a hypergraph with two node classes $\{A,B\}$ and hyperedges of a fixed size k. For $t\in\{0,1,2,\ldots,k\}$, m_t represents the number of hyperedges in H where exactly t out of the k nodes in the hyperedge come from class A. As before, $\mathbf{h}_t(A)$ and $\mathbf{h}_t(B)$ denote the type-t

affinity scores for classes A and B respectively, and can both be expressed in terms of absolute hyperedge counts:

$$\mathbf{h}_t(A) = \frac{tm_t}{\sum_{i=1}^{k} i \cdot m_i} \tag{23}$$

$$\mathbf{h}_t(B) = \frac{tm_{k-t}}{\sum_{i=1}^{k} i \cdot m_{k-i}}. \tag{24}$$

Our results apply to a generalized notion of baseline scores.

Definition. *We will refer to the set of baseline scores* $\{\boldsymbol{b}_t(X) \colon t \in [k], X \in \{A, B\}\}$ *as* realizable *or* generalized *baseline scores if they satisfy the following two assumptions:*

- *For* $t \in \{1, 2, \ldots, k\}$*,* $\boldsymbol{b}_t(A) > 0$ *and* $\boldsymbol{b}_t(B) > 0$*.*
- *There exists some two-class,* k*-uniform hypergraph* G *such that for each* $t \in \{1, 2, \ldots, k\}$*,* $\boldsymbol{b}_t(A)$ *and* $\boldsymbol{b}_t(B)$ *are the type-*t *affinity scores for classes* A *and* B *in* G*.*

As long as $\min\{|A|, |B|\} \geq k$, the standard baseline scores satisfy the above definition, by Proposition 1. We now recall the definition of majority homophily presented in the main text.

Definition. *Class* $X \in \{A, B\}$ *exhibits majority homophily if for all* $t > k - t$*,* $\boldsymbol{h}_t(X) > \boldsymbol{b}_t(X)$*.*

2.1 Impossibility Result for Odd k

Recall from the main text that when k is odd, requiring both classes to exhibit majority homophily induces a constraint on each hyperedge count m_t for $t \in \{0, 1, 2, \ldots k\}$. This is due to the fact that type-(A, t) hyperedges (t nodes from class A) are also type-$(B, k-t)$ hyperedges ($k-t$ nodes from class B). Applying a few steps of algebra, we can show that $\mathbf{h}_t(A) > \mathbf{b}_t(A)$ for $t > k - t$ implies that

$$m_t > \frac{\mathbf{b}_t(A)}{t \cdot (1 - \mathbf{b}_t(A))} \sum_{i \neq t} im_i \qquad \text{for } t = k, k-1, k-2, \ldots, (k+1)/2 \tag{25}$$

while $\mathbf{h}_t(B) > \mathbf{b}_t(B)$ for $t > k - t$ implies that

$$m_{k-t} > \frac{\mathbf{b}_t(B)}{t \cdot (1 - \mathbf{b}_t(B))} \sum_{i \neq t} im_{k-i} \qquad \text{for } t = k, k-1, k-2, \ldots, (k+1)/2 \tag{26}$$

$$\implies m_s > \frac{\mathbf{b}_{k-s}(B)}{(k-s) \cdot (1 - \mathbf{b}_{k-s}(B))} \sum_{i \neq k-s} im_{k-i} \qquad \text{for } s = 0, 1, 2, \ldots, (k-1)/2. \tag{27}$$

If there existed a type j such that m_j were not bounded below, we could set $m_j = 0$ and instead make all other hyperedge counts higher than would be expected at random, and in doing so make most hyperedge types overexpressed relative to the baseline. We will show that this is not possible when m_t is lower bounded for all $t \in \{0, 1, 2, \ldots, k\}$ as shown above. However, it is not immediately clear why lower bounds for each hyperedge type cannot all be satisfied simultaneously, especially given that half of the constraints depend on baseline scores for class A, while the other bounds depend on baseline scores for class B, which might be very different.

Proposition 6 in the main text highlights one key challenge in proving that majority homophily cannot be satisfied by two classes at once.

Proposition 6. *If any inequality from* (25) *and* (27) *is discarded, it is possible to construct a two-class k-uniform hypergraph satisfying the remaining inequalities.*

To prove this result, it will be convenient to consider the linear program (LP) that we will use to prove our impossibility results for majority homophily. A proof of Proposition 6 will follow by considering what happens in the case of the LP obtained by removing one constraint.

Linear program for measuring homophily The following linear program encodes the maximum amount of homophily that can be satisfied by classes A and B simultaneously.

$$\begin{array}{lll} \text{maximize} & \gamma & \\ \text{subject to} & t \cdot x_t - \mathbf{b}_t(A) \cdot \sum_{i=1}^{k} i \cdot x_i \geq \gamma & \text{for } t \in [k], t > k - t \\ & t \cdot x_{k-t} - \mathbf{b}_t(B) \cdot \sum_{i=1}^{k} i \cdot x_{k-i} \geq \gamma & \text{for } t \in [k], t > k - t \\ & \sum_{i=0}^{k} x_i = 1 & \\ & x_i \geq 0 & \text{for all } i \in \{0\} \cup [k] \end{array} \tag{28}$$

Recall from the main text that there is a variable $x_t \geq 0$ for each type of hyperedge in some k-uniform hypergraph. More specifically, the constraint $\sum_{i=1}^{k} x_i = 1$ encodes the fact that x_t represents the proportion of hyperedges that are of type-t (i.e., t out of k nodes are from class A). The constraint

$$t \cdot x_t - \mathbf{b}_t(A) \cdot \sum_{i=1}^{k} i \cdot x_i \geq \gamma$$

can be rearranged into the inequality:

$$\frac{t \cdot x_t}{\sum_{i=1}^{k} i \cdot x_i} \geq \mathbf{b}_t(A) + \frac{\gamma}{\sum_{i=1}^{k} i \cdot x_i}.$$

This constrains the hypergraph type-t affinity score for class A to be larger than its baseline score by at least an additive term $\gamma / \sum_{i=1}^{k} i \cdot x_i$, which will be positive if and only if γ is positive. The second set of constraints encodes similar bounds for the affinity scores of class B. A feasible solution with $\gamma = 0$ can always be achieved if the x_i variables represent hyperedge counts for a hypergraph whose affinity scores are equal to the generalized baseline scores. The following lemma shows that if the constraints are satisfied for some $\gamma > 0$, this means there exists a two-class k-uniform hypergraph where both classes exhibit majority homophily.

Lemma 7. *Let γ^* be the optimal solution to the linear program in* (28)*. There exists a two-class k-uniform hypergraph $\mathcal{H}$ with both classes exhibiting majority homophily if and only if $\gamma^* > 0$.*

Proof. Given a hypergraph where both classes satisfy majority homophily, let $x_i = m_i/M$, where M is the total number of hyperedges and m_i is the number of type-i hyperedges. The type-t affinity for class A is given by

$$\frac{t \cdot m_t}{\sum_{i=1}^{k} i \cdot m_i} = \frac{t \cdot x_t}{\sum_{i=1}^{k} i \cdot x_i}. \tag{29}$$

A similar expression in terms of x_i variables can be shown for affinity scores for class B. Choose the maximum value of γ so that all constraints are still satisfied. Since both classes are assumed to satisfy majority homophily, this γ will be strictly greater than zero.

If on the other hand we assume that $\gamma^* > 0$, the variable x_i will represent the proportion of type-i hyperedges in some two-class hypergraph where both classes exhibit majority homophily. All coefficients in the LP are rational, so its solution will be rational as well. We can therefore scale the x_i variables by a

common denominator C so that the value $M_i = Cx_i$ is an integer. To construct the appropriate hypergraph, generate M_i hyperedges of type-i for each $i \in \{0, 1, 2, \dots, k\}$. This can always be done by generating hyperedges that are completely disjoint. If one desires a specific balance between the number of nodes in classes A and B, isolated nodes from either class can be added. The resulting hypergraph provides the desired example. □

Proof of Proposition 6 Observe that Proposition 6 is equivalent to stating that if we alter the above LP by removing any one of the constraints of the form

$$tx_t - \mathbf{b}_t(A) \sum_{i=1}^{k} ix_i \geq \gamma,$$

or any constraint of the form

$$tx_{k-t} - \mathbf{b}_t(B) \sum_{i=1}^{k} ix_{k-i} \geq \gamma,$$

then the optimal solution to the resulting LP will be strictly greater than zero. We now show why this is true.

Let $r = (k+1)/2$. If $\gamma = 0$, these constraints are all satisfied tightly by setting $\tilde{x}_i = m_i/M$, where m_i is the number of type-i hyperedges and M is the total number of hyperedges, in the hypergraph whose affinity scores equal the given generalized baseline scores. Define

$$\begin{aligned} D_A &= \sum_{i=1}^{k} i\tilde{x}_i \\ D_B &= \sum_{i=1}^{k} i\tilde{x}_{k-i} = \sum_{i=0}^{k-1} (k-i)\tilde{x}_i. \end{aligned}$$

We then have

$$t\tilde{x}_t = \mathbf{b}_t(A) D_A \quad \text{for } t \in \{k, k-1, \dots, r\} \tag{30}$$

$$(k-t)\tilde{x}_t = \mathbf{b}_{k-t}(B) D_B \quad \text{for } t \in \{r-1, r-2, \dots, 2, 1\}. \tag{31}$$

Satisfying the LP constraints for some $\gamma > 0$ is equivalent to satisfying the following set of strict inequalities,

one for each of the x_i variables:

$$
\begin{aligned}
kx_k &> \mathbf{b}_k(A)\sum_{i=1}^{k} ix_i \\
(k-1)x_{k-1} &> \mathbf{b}_{k-1}(A)\sum_{i=1}^{k} ix_i \\
&\vdots \\
rx_r &> \mathbf{b}_r(A)\sum_{i=1}^{k} ix_i \\
rx_{r-1} &> \mathbf{b}_r(B)\sum_{i=0}^{k-1}(k-i)x_i \\
&\vdots \\
(k-1)x_1 &> \mathbf{b}_{k-1}(B)\sum_{i=0}^{k-1}(k-i)x_i \\
kx_0 &> \mathbf{b}_k(B)\sum_{i=0}^{k-1}(k-i)x_i.
\end{aligned}
$$

If we remove the constraint associated with variable x_t for some $t \in \{1, 2, \ldots, k-1\}$, we can satisfy the remaining inequalities strictly by setting $x_t = 0$ and keeping all other variables the same: $x_i = \tilde{x}_i = m_i/M$ for $i \neq t$. In this case, the right hand side of the equalities in (30) and (31) will strictly decrease, but the left hand side will not change. This set of variables must afterwards be normalized to sum to one, to ensure feasibility for the LP, but this does not change the fact that all inequalities (except the one we discarded) are satisfied strictly.

If we remove the constraint associated with x_0 or x_k, the proof is similar, but is slightly more involved since the first r inequalities do not involve x_0 and the second set of r inequalities do not involve x_k. We will prove the result when discarding the inequality that lower bounds x_0. By symmetry, the result holds in the same way if we removed the inequality for x_k.

After removing the inequality for x_0, consider $\varepsilon > 0$ and the following set of new variables:

$$
x_t = \begin{cases} \tilde{x}_t + \frac{\varepsilon}{t} & \text{if } t \in \{k, k-1, \ldots, r\} \\ \tilde{x}_t - \frac{\varepsilon}{t} & \text{if } t \in \{r-1, r-2, \ldots, 2, 1\} \\ 0 & \text{if } t = 0. \end{cases}
$$

For this new set of variables, we have

$$
\sum_{i=1}^{k} ix_i = \sum_{i=1}^{k} i\tilde{x}_i + \sum_{i=r}^{k} i\frac{\varepsilon}{i} - \sum_{i=1}^{r-1} i\frac{\varepsilon}{i} = D_A + \varepsilon.
$$

Also,

$$
\sum_{i=0}^{k-1}(k-i)x_i = \sum_{i=1}^{k-1}(k-i)\tilde{x}_i + \sum_{i=r}^{k-1}(k-i)\frac{\varepsilon}{i} - \sum_{i=1}^{r-1}(k-i)\frac{\varepsilon}{i} - k\tilde{x}_0 = D_B - c\varepsilon - k\tilde{x}_0,
$$

where $c = \sum_{i=1}^{r-1} \frac{k-i}{i} - \sum_{i=r}^{k-1} \frac{k-i}{i}$ is a positive constant. Observe that the first set of r constraints, which are associated with class A and variables x_k to x_r, are satisfied strictly with the new set of variables. For $t \in \{k, k-1, \dots, r\}$, we have:

$$tx_t = t\tilde{x}_t + \varepsilon > t\tilde{x}_t + \mathbf{b}_t(A)\varepsilon = \mathbf{b}_t(A)D_A + \mathbf{b}_t(A)\varepsilon = \mathbf{b}_t(A)\sum_{i=1}^{k} ix_i,$$

where we have used the fact that $\mathbf{b}_t(A) < 1$. Finally, we must simply choose any $\varepsilon > 0$ small enough that the set of inequalities for the variables $x_{r-1}, x_{r-2}, \dots, x_2, x_1$ are also satisfied strictly, which is still possible since we set $x_0 = 0$. In particular, for $t \in \{r-1, r-2, \dots, 2, 1\}$, we must satisfy

$$(k-t)x_t > \mathbf{b}_{k-t}(B)\sum_{i=0}^{k-1}(k-i)x_i.$$

Substituting in the definition of $\tilde{x}_i$, this is equivalent to

$$(k-t)\tilde{x}_t - \frac{k-t}{t}\varepsilon > \mathbf{b}_{k-t}(B)(D_B - c\varepsilon - k\tilde{x}_0).$$

Using a few steps of algebra, we can see that this is true as long as

$$k\tilde{x}_0\mathbf{b}_{k-t}(B) > \varepsilon\left(\frac{k-t}{t} - \mathbf{b}_{k-t}(B)c\right).$$

The left side of the above expression is always positive. If the right side is negative for the given set of generalized baseline scores, the inequality is trivial. If the right side is positive, there exists some $\varepsilon > 0$ that is small enough to ensure the inequality holds strictly. Therefore, if we remove the LP constraint that lower bounds x_0, there exists a set of variables whose objective score is strictly positive. This in turn implies that we can remove one inequality from (23) and (24) and find a hypergraph that satisfies all of the others. This therefore proves Proposition 6.

Primal-Dual LP Formulation. We now turn our attention back to the original linear program in (28) that includes all constraints. In order to prove results about optimal solutions this LP, we first re-write it in a general form using matrix notation and compute its dual. Let $\mathbf{x} = \begin{bmatrix} x_0 & x_1 & \cdots & x_k \end{bmatrix}$ and $\mathbf{e}$ be the all ones vector, so the constraint $\sum_{i=0}^{k} x_i = 1$ is encoded by

$$\begin{bmatrix} \mathbf{e}^T & 0 \end{bmatrix} \begin{bmatrix} \mathbf{x} \\ \gamma \end{bmatrix} = 1.$$

For odd k, let $r = (k+1)/2$. Later we will show how to make adjustments to the LP when proving impossibility results for even k. We construct a matrix $\mathbf{B}$ so that the set of constraints

$$t \cdot x_{k-t} - \mathbf{b}_t(B) \cdot \sum_{i=1}^{k} i \cdot x_{k-i} \geq \gamma \text{ for } t = k, k-1, k-2, \dots, r \tag{32}$$

$$t \cdot x_t - \mathbf{b}_t(A) \cdot \sum_{i=1}^{k} i \cdot x_i \geq \gamma \text{ for } t = r, r+1, \dots, k \tag{33}$$

is encoded by

$$\begin{bmatrix} -\mathbf{B} & \mathbf{e} \end{bmatrix} \begin{bmatrix} \mathbf{x} \\ \gamma \end{bmatrix} \leq 0.$$

In order to write the constraints in this way, we carefully order the constraints in (32) and (33) based on our ordering of x_i variables in $\mathbf{x}$. The first r rows of $\mathbf{B}$ correspond to constraints in (32), starting with $t = k$ and decreasing t until $t = r$. The second set of r rows in $\mathbf{B}$ corresponds to constraints (33), starting with $t = r$ and increasing until $t = k$.

Applying standard techniques for computing the dual of a linear program, the LP from (28) and its dual linear program are then given by

Primal Linear Program

$$\begin{aligned} \max \quad & \gamma \\ \text{s.t.} \quad & \begin{bmatrix} -\mathbf{B} & \mathbf{e} \end{bmatrix} \begin{bmatrix} \mathbf{x} \\ \gamma \end{bmatrix} \leq 0 \\ & \begin{bmatrix} \mathbf{e}^T & 0 \end{bmatrix} \begin{bmatrix} \mathbf{x} \\ \gamma \end{bmatrix} = 1 \\ & \mathbf{x} \geq 0, \gamma \geq 0 \end{aligned} \tag{34}$$

Dual Linear Program

$$\begin{aligned} \min \quad & \alpha \\ \text{s.t.} \quad & \begin{bmatrix} -\mathbf{B}^T & \mathbf{e} \\ \mathbf{e} & 0 \end{bmatrix} \begin{bmatrix} \mathbf{y} \\ \alpha \end{bmatrix} \geq \begin{bmatrix} 0 \\ 1 \end{bmatrix} \\ & \mathbf{y} \geq 0 \\ & \alpha \text{ unrestricted.} \end{aligned} \tag{35}$$

When considering optimal variables for the dual LP, it will be convenient to work with a decomposed form of the matrix $\mathbf{B}$. As an example, when $k = 3$, the matrix $\mathbf{B}$ is given by

$$\mathbf{B} = \begin{bmatrix} 3(1 - \mathbf{b}_3(B)) & -2\mathbf{b}_3(B) & -\mathbf{b}_3(B) & 0 \\ -3\mathbf{b}_2(B) & 2(1 - \mathbf{b}_2(B)) & -\mathbf{b}_2(B) & 0 \\ 0 & -\mathbf{b}_2(A) & 2(1 - \mathbf{b}_2(A)) & -3\mathbf{b}_2(A) \\ 0 & -\mathbf{b}_3(A) & -2\mathbf{b}_3(A) & 3(1 - \mathbf{b}_3(A)) \end{bmatrix} \tag{36}$$

This matrix can be decomposed as follows:

$$\mathbf{B} = \begin{bmatrix} 3 & & & \\ & 2 & & \\ & & 2 & \\ & & & 3 \end{bmatrix} - \begin{bmatrix} \mathbf{b}_3(B) & & & \\ & \mathbf{b}_2(B) & & \\ & & \mathbf{b}_2(A) & \\ & & & \mathbf{b}_3(A) \end{bmatrix} \begin{bmatrix} 3 & 2 & 1 & 0 \\ 3 & 2 & 1 & 0 \\ 0 & 1 & 2 & 3 \\ 0 & 1 & 2 & 3 \end{bmatrix}. \tag{37}$$

In general for odd k, we can decompose the matrix $\mathbf{B}$ in the following way:

$$\mathbf{B} = \mathbf{D}_k - \mathbf{D}_{\mathbf{b}}\mathbf{R}, \tag{38}$$

where $\mathbf{D}_k$ is a diagonal matrix with diagonal entries $[k, k-1, \cdots, r, r+1, r+1, r, \cdots, k-1, k]$, and $\mathbf{D}_{\mathbf{b}}$ is a diagonal matrix with diagonal entries $[\mathbf{b}_k(B), \mathbf{b}_{k-1}(B), \cdots \mathbf{b}_r(B), \mathbf{b}_r(A), \cdots, \mathbf{b}_{k-1}(A), \mathbf{b}_k(A)]$. The

first r rows of matrix $\mathbf{R}$ are $\begin{bmatrix} k & k-1 & \cdots & 1 & 0 \end{bmatrix}$, and the next r rows are $\begin{bmatrix} 0 & 1 & \cdots & k-1 & k \end{bmatrix}$:

$$\mathbf{D}_k = \begin{bmatrix} k & & & & & \\ & \ddots & & & & \\ & & r & & & \\ & & & r & & \\ & & & & \ddots & \\ & & & & & k \end{bmatrix}$$

$$\mathbf{D}_{\mathbf{b}} = \begin{bmatrix} \mathbf{b}_k(B) & & & & & \\ & \ddots & & & & \\ & & \mathbf{b}_r(B) & & & \\ & & & \mathbf{b}_r(A) & & \\ & & & & \ddots & \\ & & & & & \mathbf{b}_k(A) \end{bmatrix}$$

$$\mathbf{R} = \begin{bmatrix} k & \cdots & 1 & 0 \\ \vdots & \vdots & \vdots & \vdots \\ k & \cdots & 1 & 0 \\ 0 & 1 & \cdots & k \\ \vdots & \vdots & \vdots & \vdots \\ 0 & 1 & \cdots & k \end{bmatrix}.$$

We can quickly obtain a solution with objective score of $\alpha = 0$ for the primal linear program. Recall that by assumption, the baseline scores correspond to affinity scores for some k-uniform hypergraph G. Let m_t be the number of hypergraphs of type t in G, and M be the total number of hyperedges. Then define a set of primal solutions $\mathbf{x}$ by setting $x_t = m_t/M$ for $t \in \{0, 1, 2, \ldots k\}$, and set $\gamma = 0$. The fact that the affinity score in this hypergraph equals the baseline score means that this set of primal variables is feasible for the primal LP. The following lemma, which proves our majority homophily result for odd k in Theorem 5, shows that these are in fact optimal primal solutions.

Lemma 8. *For an odd integer k and $r = (k+1)/2$, define $\delta = 2k\sum_{t=r}^{k} \frac{1}{t}$, and consider the following set of dual variables:*

$$\alpha = 0 \tag{39}$$

$$y_{B,k} = \frac{2}{\delta} \cdot \frac{\sum_{i=r}^{k}(\frac{k}{i} - 1)\boldsymbol{b}_i(B)}{1 - \sum_{i=r}^{k}(2 - \frac{k}{i})\boldsymbol{b}_i(B)} \tag{40}$$

$$y_{B,t} = \frac{2}{\delta}\left(\frac{k}{t} - 1\right) + \left(2 - \frac{k}{t}\right) y_{B,k} \text{ for } t \in \{r, \cdots, k-1\} \tag{41}$$

$$y_{A,t} = \frac{2k}{\delta t} - y_{B,t} \text{ for } t \in \{r, \cdots, k\}. \tag{42}$$

If $Y = \sum_{t=r}^{k} y_{A,t} + y_{B,t}$, then the set of normalized dual variables defined by $\tilde{y}_{X,t} = y_{X,t}/Y$ for $X \in \{A, B\}$ and $t \in \{r, \ldots, k\}$ is feasible for the dual LP for majority homophily.

Proof. When considering variables for the dual LP (35), first recall that the matrix $\mathbf{B}$ can be decomposed into the form $\mathbf{B} = \mathbf{D}_k - \mathbf{D}_{\mathbf{b}}\mathbf{R}$, where $\mathbf{D}_{\mathbf{b}}$ is a diagonal matrix with diagonal entries

$$[\mathbf{b}_k(B), \mathbf{b}_{k-1}(B), \cdots, \mathbf{b}_r(B), \mathbf{b}_r(A), \cdots, \mathbf{b}_{k-1}(A), \mathbf{b}_k(A)].$$

In this way, each row and column of $\mathbf{B}$ can be mapped to a pair (X, t) where $t \in \{r, r+1, \dots, k\}$ represents a hyperedge type and $X \in \{A, B\}$ is a class. Therefore, each dual variable is also associated with an (X, t) pair, which is why we doubly-index dual variables in $\mathbf{y}$ as follows:

$$\mathbf{y}^T = \begin{bmatrix} y_{B,k} & y_{B,k-1} & \cdots & y_{B,r} & y_{A,r} & \cdots & y_{A,k-1} & y_{A,k} \end{bmatrix}.$$

In the remainder of the proof, we will show that the unnormalized dual variables given in the lemma statement are nonnegative and satisfy $\mathbf{B}^T\mathbf{y} = 0$. In their current form, these variables do not satisfy $\mathbf{e}^T\mathbf{y} = 1$, but this can easily be fixed by dividing the entries of $\mathbf{y}$ by their sum to produce a vector $\hat{\mathbf{y}}$ whose entries sum to 1. At this point, the vector $\hat{\mathbf{y}}$ along with $\alpha = 0$ provides a feasible solution with objective score of zero for the dual LP, which will conclude the proof. Thus, in the remainder of the proof we prove that the variables in (40), (41), and (42) are nonnegative and satisfy $\mathbf{B}^T\mathbf{y} = 0$.

Nonnegativity of dual variables. The nonnegativity of dual variables follows from the nonnegativity of baseline scores. Note in particular that

$$\sum_{i=r}^{k}(2 - k/i)\mathbf{b}_i(B) \leq \sum_{i=r}^{k}\mathbf{b}_i(B) < 1$$

which shows that the denominator of $y_{B,k}$ is positive. The numerator is also positive by inspection. Since $y_{B,k} > 0$, we can see that all three terms in $y_{B,t}$ are positive. Finally,

$$\begin{aligned} y_{A,t} &= \frac{2}{\delta} - \left(2 - \frac{k}{t}\right) y_{B,k} \geq \frac{2}{\delta} - y_{B,k} \\ &= \frac{2}{\delta}\left(1 - \frac{\sum_{i=r}^{k}(\frac{k}{i} - 1)\mathbf{b}_i(B)}{1 - \sum_{i=r}^{k}(2 - \frac{k}{i})\mathbf{b}_i(B)}\right) \\ &= \frac{2}{\delta}\left(\frac{1 - \sum_{i=r}^{k}(2 - \frac{k}{i})\mathbf{b}_i(B) - \sum_{i=r}^{k}(\frac{k}{i} - 1)\mathbf{b}_i(B)}{1 - \sum_{i=r}^{k}(2 - \frac{k}{i})\mathbf{b}_i(B)}\right) \\ &= \frac{2}{\delta}\left(\frac{1 - \sum_{i=r}^{k}\mathbf{b}_i(B)}{1 - \sum_{i=r}^{k}(2 - \frac{k}{i})\mathbf{b}_i(B)}\right). \end{aligned}$$

As before, the numerator and denominator are both positive, so $y_{A,t} > 0$.

Proving $\mathbf{B}^T\mathbf{y} = 0$. Given the decomposition $\mathbf{B} = \mathbf{D}_k - \mathbf{D}_\mathbf{b}\mathbf{R}$, we can see that proving $\mathbf{B}^T\mathbf{y} = 0$ is equivalent to showing

$$\mathbf{D}_k\mathbf{y} = \mathbf{R}^T\mathbf{D}_\mathbf{b}\mathbf{y}. \tag{43}$$

If we doubly index entries of $\mathbf{D}_k\mathbf{y}$ using the same indexing as the $\mathbf{y}$ entries, we see that

$$[\mathbf{D}_k\mathbf{y}]_{B,t} = t y_{B,t}.$$

Meanwhile, the right hand side of (43) is

$$\mathbf{R}^T\mathbf{D}_\mathbf{b}\mathbf{y} = \begin{bmatrix} k & k & \cdots & k & 0 & \cdots & 0 \\ k-1 & k-1 & \cdots & k-1 & 1 & \cdots & 1 \\ \vdots & \vdots & \ddots & \vdots & \vdots & \ddots & \vdots \\ 1 & 1 & \cdots & 1 & k-1 & \cdots & k-1 \\ 0 & 0 & \cdots & 0 & k & \cdots & k \end{bmatrix} \begin{bmatrix} y_{B,k}\mathbf{b}_k(B) \\ y_{B,k-1}\mathbf{b}_{k-1}(B) \\ \vdots \\ y_{B,r}\mathbf{b}_r(B) \\ y_{A,r}\mathbf{b}_r(A) \\ \vdots \\ y_{A,k}\mathbf{b}_k(A) \end{bmatrix}$$

After canceling k from both sides, the first row of the matrix equation (43) is:

$$\begin{aligned} y_{B,k} = \sum_{i=r}^{k} y_{B,i}\mathbf{b}_i(B) &= \sum_{i=r}^{k}\left[\frac{2}{\delta}\left(\frac{k}{i}-1\right)+\left(2-\frac{k}{i}\right)y_{B,k}\right]\mathbf{b}_i(B) \\ \iff y_{B,k}\left(1-\sum_{i=r}^{k}(2-k/i)\mathbf{b}_i(B)\right) &= \frac{2}{\delta}\sum_{i=r}^{k}(k/i-1)\mathbf{b}_i(B) \\ \iff y_{B,k} &= \frac{2}{\delta}\cdot\frac{\sum_{i=r}^{k}(\frac{k}{i}-1)\mathbf{b}_i(B)}{1-\sum_{i=r}^{k}(2-\frac{k}{i})\mathbf{b}_i(B)}. \end{aligned}$$

So this holds by the definition of $y_{B,k}$ in (40). Next, we show $[\mathbf{D}_k\mathbf{y}]_{B,t} = [\mathbf{R}^T\mathbf{D}_b\mathbf{y}]_{B,t}$ for $t \in \{r,\ldots,k-1\}$. Let $y = y_{B,k} = \sum_{i=r}^{k}\mathbf{b}_i(B)y_{B,i}$. Each such equation has the form

$$\begin{aligned} \frac{2t}{\delta}(\frac{k}{t}-1)+(2-\frac{k}{t})yt &= t\sum_{i=r}^{k}\mathbf{b}_i(B)y_{B,i}+(k-t)\sum_{i=r}^{k}\mathbf{b}_i(A)y_{A,i} \\ \iff \frac{2}{\delta}(k-t)+(2t-k)y &= ty+(k-t)\sum_{i=r}^{k}\mathbf{b}_i(A)y_{A,i} \\ \iff \frac{2}{\delta}-y &= \sum_{i=r}^{k}\mathbf{b}_i(A)\left(\frac{2}{\delta}-(2-\frac{k}{i})y\right) \\ \iff \frac{2}{\delta}\left(1-\sum_{i=r}^{k}\mathbf{b}_i(A)\right) &= y\left(1-\sum_{i=r}^{k}\mathbf{b}_i(A)(2-\frac{k}{i})\right) \\ \iff y &= \frac{2}{\delta}\frac{1-\sum_{i=r}^{k}\mathbf{b}_i(A)}{1-\sum_{i=r}^{k}\mathbf{b}_i(A)(2-\frac{k}{i})}. \end{aligned}$$

Therefore, the equivalence between the first r entries in the equation (43) will hold as long as we can prove that the following are both equivalent ways of writing $y = y_{B,k}$:

$$y = y_{B,k} = \frac{2}{\delta}\frac{1-\sum_{i=r}^{k}\mathbf{b}_i(A)}{1-\sum_{i=r}^{k}\mathbf{b}_i(A)(2-\frac{k}{i})} = \frac{2}{\delta}\frac{\sum_{i=r}^{k}(\frac{k}{i}-1)\mathbf{b}_i(B)}{1-\sum_{i=r}^{k}(2-\frac{k}{i})\mathbf{b}_i(B)}. \tag{44}$$

We can prove this by using the fact that there is a hypergraph G whose affinity scores equal the baseline scores. More specifically, for $i \in [k]$,

$$\begin{aligned} \mathbf{b}_i(A) &= \frac{im_i}{D_A} \\ \mathbf{b}_i(B) &= \frac{im_{k-i}}{D_B}, \end{aligned}$$

where $D_A = \sum_{i=1}^{k} im_i$ and $D_B = \sum_{i=1}^{k} im_{k-i}$. Re-writing the numerator and denominator of the first ratio in (44) (and after scaling each expression by $\delta/2$) we get

$$\frac{1-\sum_{i=r}^{k}\mathbf{b}_i(A)}{1-\sum_{i=r}^{k}\mathbf{b}_i(A)(2-\frac{k}{i})} = \frac{\frac{1}{D_A}\sum_{i=1}^{r-1} im_i}{1-\frac{1}{D_A}\sum_{i=r}^{k} m_i(2i-k)} = \frac{\sum_{i=1}^{r-1} im_i}{D_A-\sum_{i=r}^{k} m_i(2i-k)}.$$

Similarly, we re-write the second ratio:

$$\frac{\sum_{i=r}^{k}(\frac{k}{i}-1)\mathbf{b}_i(B)}{1-\sum_{i=r}^{k}(2-\frac{k}{i})\mathbf{b}_i(B)} = \frac{\frac{1}{D_B}\sum_{i=r}^{k}(k-i)m_{k-i}}{1-\frac{1}{D_B}\sum_{i=r}^{k}(2i-k)m_{k-i}} = \frac{\sum_{i=r}^{k}(k-i)m_{k-i}}{D_B-\sum_{i=r}^{k}(2i-k)m_{k-i}}.$$

Written this way, we see that the numerators are the same since

$$\sum_{i=r}^{k}(k-i)m_{k-i} = \sum_{t=1}^{r-1} t m_t. \tag{45}$$

It remains to show that the denominators are equal, which we prove by considering a sequence of equivalent statements:

$$\begin{aligned}
D_B - \sum_{i=r}^{k}(2i-k)m_{k-i} &= D_A - \sum_{i=r}^{k} m_i(2i-k) \\
\iff \sum_{i=1}^{k} i m_{k-i} - \sum_{i=r}^{k}(2i-k)m_{k-i} &= \sum_{i=1}^{k} i m_i - \sum_{i=r}^{k} m_i(2i-k) \\
\iff \sum_{i=0}^{k} i m_{k-i} - \sum_{i=0}^{k} i m_i &= \sum_{i=r}^{k}(2i-k)m_{k-i} - \sum_{i=r}^{k} m_i(2i-k) \\
\iff \sum_{i=0}^{k}[(k-i)-i]m_i &= -\sum_{j=0}^{r-1}(2j-k)m_j - \sum_{i=r}^{k} m_i(2i-k) \\
\iff \sum_{i=0}^{k}(k-2i)m_i &= \sum_{i=0}^{k}(k-2i)m_i.
\end{aligned}$$

At this point our proof has shown that the first r rows (the rows corresponding to class B), of the matrix equation $\mathbf{D}_k\mathbf{y} = \mathbf{R}^T\mathbf{D}_\mathbf{b}\mathbf{y}$ hold. We use a similar approach to show the remaining r rows also hold. First note that for $t \in \{r, r+1, \dots, k\}$,

$$[\mathbf{D}_k\mathbf{y}]_{A,t} = t\cdot(y_{A,t}) = t\cdot\left(\frac{2}{\delta} - \left(2-\frac{k}{t}\right)y_{B,k}\right).$$

The last entry of the equation is $[\mathbf{D}_k\mathbf{y}]_{A,k} = [\mathbf{R}^T\mathbf{D}_\mathbf{b}\mathbf{y}]_{A,k}$, which holds by the following sequence of equivalent statements:

$$\begin{aligned}
(2/\delta - y_{B,k}) &= \sum_{i=r}^{k} y_{A,i}\mathbf{b}_i(A) \\
\iff (2/\delta - y_{B,k}) &= \sum_{i=r}^{k}\mathbf{b}_i(A)\left(\frac{2}{\delta} - \left(2-\frac{k}{i}\right)y_{B,k}\right) \\
\iff \frac{2}{\delta}\quad 1-\sum_{i=r}^{k}\mathbf{b}_i(A)\Bigg) &= y_{B,k}\quad 1-\sum_{i=r}^{k}(2-\frac{k}{i})\mathbf{b}_i(A)\Bigg).
\end{aligned}$$

The last equation holds by the equivalent ways of writing $y_{B,k}$ shown in (44).

Finally, we confirm that $[\mathbf{D}_k\mathbf{y}]_{A,t} = [\mathbf{R}^T\mathbf{D}_\mathbf{b}\mathbf{y}]_{A,t}$ holds for $t \in \{r, \dots, k-1\}$. Let $y = y_{B,k}$ and recall from the last step that $2/\delta - y = \sum_{i=r}^{k} y_{A,i}\mathbf{b}_i(A)$. Each equation corresponding to one of the last r rows has the form

$$
\begin{aligned}
& t \cdot \left(\frac{2}{\delta} - \left(2 - \frac{k}{t}\right) y\right) = t\sum_{i=r}^{k} \mathbf{b}_i(A) y_{A,i} + (k-t)\sum_{i=r}^{k} \mathbf{b}_i(B) y_{B,i} \\
\iff & \left(\frac{2t}{\delta} - (2t-k)\, y\right) = \frac{2t}{\delta} - yt + (k-t)\sum_{i=r}^{k} \mathbf{b}_i(B) y_{B,i} \\
\iff & -(t-k)y = (k-t)\sum_{i=r}^{k} \mathbf{b}_i(B) y_{B,i} \\
\iff & y = \sum_{i=r}^{k} \mathbf{b}_i(B) y_{B,i},
\end{aligned}
$$

which again was shown in previous steps. At this point we have shown that all entries in the equation $\mathbf{D}_k\mathbf{y} = \mathbf{R}^T\mathbf{D}_\mathbf{b}\mathbf{y}$ hold. Therefore, the dual variables are nonnegative and satisfy $\mathbf{B}^T\mathbf{y} = 0$, concluding the proof. □

2.2 Proof for even k

When k is even, the definition of majority homophily does not place any restriction on type-$(k/2)$ hyperedges for either class, since neither class is strictly in the majority for these hyperedges. If the number of type-$(k/2)$ hyperedges is small enough, it is possible for both classes to exhibit majority homophily. As one example, starting with a complete hypergraph and deleting all hyperedges of type-$(k/2)$ will produce a hypergraph where both classes satisfy majority homophily. However, we can still prove an analogous impossibility result for even k if we add one extra constraint. We restate and prove our result for even k.

Theorem 5, even k. *When k is even, it is impossible for both classes A and B to exhibit majority homophily if additionally $\boldsymbol{h}_\ell(A) > \boldsymbol{b}_\ell(A)$ or $\boldsymbol{h}_\ell(B) > \boldsymbol{b}_\ell(B)$ for $\ell = k/2$.*

Proof. The proof follows the same steps as the proof for odd k, with minor alterations to the linear program and the optimal dual variables. We highlight key changes that must be made and for brevity skip steps that are nearly identical to the proof of the previous result.

Let $\ell = k/2$. Without loss of generality we prove the result is impossible if we restrict $\mathbf{h}_\ell(A) > \mathbf{b}_\ell(A)$. By symmetry, the same impossibility result holds if we added the new constraint for class B instead. We begin by altering the LP from (28) to include an additional constraint:

$$
\ell \cdot x_\ell - \mathbf{b}_t(A) \cdot \sum_{i=1}^{k} i \cdot x_i \geq \gamma. \tag{46}
$$

For this new linear program, we can again confirm that the optimal score γ^* will be greater than zero if and only if it is possible for both classes to exhibit majority homophily *and* for constraint (46) to hold. We then can again re-write the LP and its dual in the form shown in (34) and (35), by extending the matrix $\mathbf{B}$ to include one extra row to account for the new constraint. By our assumptions about baseline scores, we know there exists a hypergraph G, with M total hyperedges and m_i hyperedges of type-i, such that the affinity scores of G equal the baseline scores in question. A primal feasible solution with an objective score of zero can then be realized by setting $x_i = m_i/M$ and $\gamma = 0$.

Next, let $r = k/2 + 1$ and construct the following set of dual variables:

$$y_{B,k} = \frac{2}{\delta} \cdot \frac{\sum_{i=r}^{k} (\frac{k}{i} - 1)\mathbf{b}_i(B)}{1 - \sum_{i=r}^{k} (2 - \frac{k}{i})\mathbf{b}_i(B)}$$

$$y_{B,t} = \frac{2}{\delta}\left(\frac{k}{t} - 1\right) + \left(2 - \frac{k}{t}\right) y_{B,k} \quad \text{for } t \in \{r, \cdots, k-1\}$$

$$y_{A,t} = \frac{2k}{\delta t} - y_{B,t} \quad \text{for } t \in \{r, \cdots, k\}$$

$$y_{A,\ell} = 2/\delta,$$

where $\delta = 2 + 2k \sum_{t=r}^{k} \frac{1}{t}$. The result again relies on the fact that $y_{B,k}$ can be written in two ways, using the fact that baseline scores correspond to affinity scores for some hypergraph G:

$$y_{B,k} = \frac{2}{\delta} \frac{1 - \sum_{i=\ell}^{k} \mathbf{b}_i(A)}{1 - \sum_{i=\ell}^{k} \mathbf{b}_i(A)(2 - \frac{k}{i})} = \frac{2}{\delta} \frac{\sum_{i=r}^{k} (\frac{k}{i} - 1)\mathbf{b}_i(B)}{1 - \sum_{i=r}^{k} (2 - \frac{k}{i})\mathbf{b}_i(B)}.$$

Using the same basic set of steps used in Lemma 8, we can show that $\mathbf{B}^T \mathbf{y} = 0$ and $\mathbf{y} \geq 0$. Scaling the variables to sum to one produces a dual feasible solution with an objective score of zero, which proves the result. □

2.3 Impossibility Results for Normalized Bias Scores

One alternative approach to measuring an affinity score's deviation from baseline is to consider the normalized bias score introduced in the main text. For a class X, the type-t normalized bias score is

$$\mathbf{f}_t(X) = \begin{cases} \frac{\mathbf{h}_t(X) - \mathbf{b}_t(X)}{1 - \mathbf{b}_t(X)} & \text{if } \mathbf{h}_t(X) \geq \mathbf{b}_t(X) \\ \\ \frac{\mathbf{h}_t(X) - \mathbf{b}_t(X)}{\mathbf{b}_t(X)} & \text{if } \mathbf{h}_t(X) < \mathbf{b}_t(X). \end{cases} \quad (47)$$

Our existing notion of strict majority homophily is equivalent to requiring $\mathbf{f}_t(X) > 0$ whenever $t > k/2$. We see therefore that the same impossibility results and combinatorial limits apply to a natural notion of majority homophily for normalized bias scores. The natural way to define strict monotonic homophily for normalized bias scores is to require $\mathbf{f}_t(X) > \mathbf{f}_{t-1}(X)$ whenever $t > k/2$, which can be different from monotonicity of ratio scores. Nevertheless, in our empirical results we find that ratio scores and normalized bias scores often increase and decrease in similar patterns. Furthermore, we can prove the same type of impossibility results for normalized bias scores by adding one natural assumption regarding the balance in homophily levels exhibited by two node classes.

Theorem 9. *Let $H = (V, E)$ be a two-class k-uniform hypergraph and $\{\boldsymbol{b}_i(X) \colon i \in [k], X \in \{A, B\}\}$ be realizable baseline scores. Let k be odd and $r = (k+1)/2$. If $\boldsymbol{f}_r(A)$ and $\boldsymbol{f}_r(B)$ have the same sign, then it is impossible for both A and B to simultaneously exhibit strict monotonic homophily in terms of normalized bias scores.*

Proof. If we consider first of all the case where $\mathbf{f}_r(A) > 0$ and $\mathbf{f}_r(B) > 0$, assuming that normalized bias scores are strictly increasing for both classes implies that $\mathbf{f}_t(X) > 0$ whenever $t > k/2$ for each $X \in \{A, B\}$. This would mean that both classes satisfy strict majority homophily, which is impossible. If on the other hand we have $\mathbf{f}_r(A) \leq 0$ and $\mathbf{f}_r(B) \leq 0$, then $\mathbf{f}_r(X) > \mathbf{f}_{r-1}(X)$ means that

$$\frac{\mathbf{h}_r(X) - \mathbf{b}_r(X)}{\mathbf{b}_r(X)} > \frac{\mathbf{h}_{r-1}(X) - \mathbf{b}_{r-1}(X)}{\mathbf{b}_{r-1}(X)} \implies \frac{\mathbf{h}_r(X)}{\mathbf{b}_r(X)} > \frac{\mathbf{h}_{r-1}(X)}{\mathbf{b}_{r-1}(X)}.$$

Assuming that this holds for both classes at once is again a contradiction, as shown in the proof of Theorem 3. □

We can similarly prove impossibility results for even values of k by adding an additional assumption as we did in Theorem 4. The assumption that $\mathbf{f}_r(A)$ and $\mathbf{f}_r(B)$ share the same sign is in line with the goal of trying to understand whether two classes can satisfy the same homophily properties at the same time. We conjecture that Theorem 9 holds without this assumption, though we leave a more in depth analysis for future work. In any case, this theorem confirms that even if there is some way for both classes to have strictly increasing normalized bias scores (which there may not be), there will still be a fundamental imbalance in their affinity scores and in the level of homophily they exhibit. This further confirms the message that natural notions of group homophily are governed by subtle combinatorial limits that must exist independent of human preferences and choices.

3 Derivation and Results for Alternative Affinity Scores

The hypergraph affinity scores we consider in the main text and the previous two sections of the supplement are based on ratios of typed degrees for nodes in a certain class. This directly generalizes the standard approach that has been used for measuring homophily in graphs [16]. Another natural approach is to consider affinity scores defined by ratios of hyperedge types. Formally, given the same k-uniform hypergraph $H = (V, E)$ where m_t denotes the number of type-t hyperedges, we can measure the following *alternative* affinity scores:

$$\mathbf{a}_t(A) = \frac{m_t}{\sum_{i=1}^k m_i} \tag{48}$$

$$\mathbf{a}_t(B) = \frac{m_{k-t}}{\sum_{i=1}^k m_{k-i}}. \tag{49}$$

For each class $X \in \{A, B\}$, these ratios directly measure the proportion of hyperedges of type-(X, t), among all hyperedges involving at least one class X node. In this section we show that all of our main theoretical results also hold for these alternative scores. This first of all highlights that our main results on hypergraph homophily are broadly true for a wide range of notions of affinity scores. Furthermore, as we shall also see, our main impossibility results are in fact easier to show for these alternative scores, and our proof that these scores correspond to maximum likelihood estimates of a certain model parameter is more direct and does not require approximations.

3.1 Baseline Scores for Alternative Affinities

Analogous to our approach for standard affinity scores, we define the baseline score for $\mathbf{a}_t(X)$ to be the probability that we obtain a type-(X, t) hyperedge if we select a k-tuple uniformly at random from among all k-tuples involving at least one X node:

$$\mathbf{b}_t(X) = \frac{\binom{|X|}{t}\binom{N-|X|}{k-t}}{\sum_{i=1}^k \binom{|X|}{i}\binom{N-|X|}{k-i}}. \tag{50}$$

The denominator counts all k-tuples involving at least one node from X. For simplicity, we use the same notation as we did for standard affinity scores.

Lemma 10. *Let $H = (V, E)$ be a complete k-uniform hypergraph with two node classes $\{A, B\}$. For $t \in [k]$, the type-t alternative affinity scores for class $X \in \{A, B\}$ equals the type-t alternative baseline score (50): $\mathbf{a}_t(X) = \boldsymbol{b}_t(X)$.*

The proof of this lemma is omitted as it follows the same steps as Lemma 1. We will prove homophily impossibility results for the following more general notion of baseline scores.

Definition. *Let k be a fixed constant. The set of scores $\{\boldsymbol{b}_t(X) \colon X \in \{A, B\}, t \in [k]\}$ are generalized baseline scores for alternative affinity scores $\{\mathbf{a}_t(X) \colon X \in \{A, B\}, t \in [k]\}$ if the following two conditions hold:*

- $\boldsymbol{b}_t(X) > 0$ *for* $X \in \{A, B\}$ *and* $t \in [k]$.
- *The scores* $\{\boldsymbol{b}_t(X)\}$ *correspond to alternative affinity scores for some k-uniform hypergraph G with two node classes.*

3.2 Interpreting Scores as Maximum Likelihood Estimates

We can interpret the alternative affinity score $\mathbf{a}_t(A)$ as the maximum likelihood estimate for a certain affinity parameter of a binomial distribution for hyperedge data. An analogous interpretation also applies for class B. We begin by considering a slight variation of the cardinality-based HSBM. This new model still considers a set of n nodes separated into two classes $\{A, B\}$, and generates typed hyperedges based on a set of probabilities $\mathbf{p} = \begin{bmatrix} p_0 & p_1 & \cdots & p_k \end{bmatrix}$. Let $\mathcal{K}_t$ be the set of k-tuples of type-t. For each $e \in \mathcal{K}_t$, let X_e be a Poisson random variable with parameter p_t, and let this represent the number of hyperedges placed at e:

$$X_e \sim \text{Poisson}(p_t). \tag{51}$$

Recall that the cardinality-based HSBM differs in that we instead defined $X_e \sim \text{Bernoulli}(p_t)$. When we consider $p_t = o(1)$, which will typically be the case, this Poisson distribution will be very close to a Bernoulli with parameter p_t. For a hypergraph generated from this distribution, let M_j be the random variable representing the number of hyperedges of type-j in H, which as a sum of Poisson random variables will also be Poisson:

$$M_j = \sum_{e \in \mathcal{K}_j} X_e \sim \text{Poisson}(K_j p_j). \tag{52}$$

Define M_A to be the random variable denoting the total number of hyperedges involving at least one node in A, which is also Poisson distributed:

$$M_A = \sum_{j=1}^{k} M_j \sim \text{Poisson}\left(\sum_{j=1}^{k} K_j p_j\right) \tag{53}$$

The random variable representing the number of hyperedges that are *not* type-j is given by

$$\tilde{M_j} = \sum_{i \neq j} M_i \sim \text{Poisson}\left(\sum_{i \neq j} K_i p_i\right). \tag{54}$$

Consider now a fixed hypergraph H with m_A hyperedges involving at least one class-A node. If we assume this hypergraph was drawn from the random distribution given above, the random variable M_t, conditioned on the observed hyperedge count m_A, will be binomial:

$$M_t \mid m_A \sim \text{Binom}(m_A, f_t) \tag{55}$$

where f_t is an affinity parameter:

$$f_t = \frac{K_t p_t}{\sum_{j=1}^{k} K_j p_j}. \tag{56}$$

To see why, note

$$
\begin{aligned}
\Pr(M_t = c \mid M_A = m_A) &= \frac{\Pr(M_t = m_t \text{ and } M_A = m_A)}{\Pr(M_A = m_A)} \\
&= \frac{\Pr(M_t = m_t \text{ and } \tilde{M}_j = m_A - c)}{\Pr(M_A = m_A)} \\
&= \frac{\left[\frac{1}{c!}(p_t K_t)^c \cdot e^{-p_t K_t}\right]\left[\frac{1}{(m_A - c)!}(\sum_{i \neq t} p_i K_i)^{m_A - c} \cdot e^{-\sum_{i \neq t} p_i K_i}\right]}{\frac{1}{(m_A)!}(\sum_{i=1}^k p_i K_i)^{m_A} \cdot e^{-\sum_{i=1}^k p_i K_i}} \\
&= \binom{m_A}{c}\left(\frac{K_t p_t}{\sum_{j=1}^k K_j p_j}\right)^c \left(1 - \frac{K_t p_t}{\sum_{j=1}^k K_j p_j}\right)^{m_A - c}.
\end{aligned}
$$

Finally, given an observed hypergraph with m_A hyperedges involving at least one node in A, the likelihood of observing m_t hyperedges of type-t under this model is

$$\Pr(M_t = m_t \mid M_A = m_A) = \binom{m_A}{c} f_t^{m_t}(1 - f_t)^{m_A - m_t}.$$

Taking a derivative of the log-likelihood function with respect to the parameter f_t and setting it to zero will give the maximum likelihood estimate for f_t. This ends up being equal to the alternative type-t affinity score,

$$f_t = \frac{m_t}{m_A} = \frac{m_t}{\sum_{i=1}^k m_i}.$$

3.3 Impossibility Results

Analogous to our results for standard affinity scores, we define two notions of hypergraph homophily based on alternative baseline scores. Let $\{\mathbf{b}_t(X) \colon X \in \{A, B\}, t \in [k]\}$ denote a set of generalized baseline scores.

Definition. *Class $X \in \{A, B\}$ exhibits majority homophily if for all $t > k - t$,*

$$\mathbf{a}_t(X) > \boldsymbol{b}_t(X). \tag{57}$$

Definition. *Class $X \in \{A, B\}$ exhibits monotonic homophily if for all $t > k - t$,*

$$\frac{\mathbf{a}_t(X)}{\boldsymbol{b}_t(X)} > \frac{\mathbf{a}_{t-1}(X)}{\boldsymbol{b}_{t-1}(X)}. \tag{58}$$

The proof of the following impossibility result for monotonic homophily follows the same steps as the proof for Theorem 3. In particular, these results can be shown by considering only two types of hyperedges.

Theorem 11. *Let H be a two-class, k-uniform hypergraph and $\{\boldsymbol{b}_t(X)\}$ be a set of generalized baseline scores for alternative affinity scores $\{\mathbf{a}_t(X)\}$. If k is odd, it is impossible for both classes to exhibit monotonic homophily in terms of alternative affinity scores. If k is even, then both classes can exhibit monotonic homophily, but in this case $\frac{\mathbf{a}_\ell(X)}{\boldsymbol{b}_\ell(X)} < \frac{\mathbf{a}_{\ell-1}(X)}{\boldsymbol{b}_{\ell-1}(X)}$ for $\ell = k/2$ and $X \in \{A, B\}$.*

In order to prove impossibility results for majority homophily, we use the same linear programming based proof technique that we used for Theorem 5.

Theorem 12. *Let H be a two-class, k-uniform hypergraph and $\{\boldsymbol{b}_t(X)\}$ be a set of generalized baseline scores for alternative affinity scores $\{\mathbf{a}_t(X)\}$. If k is odd, it is impossible for both classes to exhibit majority homophily in terms of alternative affinity scores.*

Proof. Let $r = (k+1)/2$. The following linear program will have a strictly positive solution if and only if it is possible for both classes to exhibit majority homophily, with respect to the new alternative affinity scores:

$$\begin{array}{lll} \text{maximize} & \gamma & \\ \text{subject to} & x_t - \mathbf{b}_t(A) \cdot \sum_{i=1}^{k} x_i \geq \gamma & \text{for } t \in [k], t > k-t \\ & x_{k-t} - \mathbf{b}_t(B) \cdot \sum_{i=1}^{k} x_{k-i} \geq \gamma & \text{for } t \in [k], t > k-t \\ & \sum_{i=0}^{k} x_i = 1 & \\ & x_i \geq 0 & \text{for all } i \in \{0\} \cup [k] \end{array} \tag{59}$$

The variable x_t again represents the proportion of hyperedges where t of the nodes are from class A. We can express the linear program as well as its dual in the same general form

Primal Linear Program

$$\begin{array}{ll} \max & \gamma \\ \text{s.t.} & \begin{bmatrix} -\mathbf{B} & \mathbf{e} \end{bmatrix} \begin{bmatrix} \mathbf{x} \\ \gamma \end{bmatrix} \leq 0 \\ & \begin{bmatrix} \mathbf{e}^T & 0 \end{bmatrix} \begin{bmatrix} \mathbf{x} \\ \gamma \end{bmatrix} = 1 \\ & \mathbf{x} \geq 0, \gamma \geq 0 \end{array} \tag{60}$$

Dual Linear Program

$$\begin{array}{ll} \min & \alpha \\ \text{s.t.} & \begin{bmatrix} -\mathbf{B}^T & \mathbf{e} \\ \mathbf{e} & 0 \end{bmatrix} \begin{bmatrix} \mathbf{y} \\ \alpha \end{bmatrix} \geq \begin{bmatrix} 0 \\ 1 \end{bmatrix} \\ & \mathbf{y} \geq 0 \\ & \alpha \text{ unrestricted.} \end{array} \tag{61}$$

Above, $\mathbf{e}$ is the all ones vector and the matrix $\mathbf{B}$ encodes constraints of the form

$$x_{k-t} - \mathbf{b}_t(B) \cdot \sum_{i=1}^{k} x_{k-i} \geq \gamma \text{ for } t = k, k-1, k-2, \dots, r \tag{62}$$

$$x_t - \mathbf{b}_t(A) \cdot \sum_{i=1}^{k} x_i \geq \gamma \text{ for } t = r, r+1, \dots, k. \tag{63}$$

The matrix $\mathbf{B}$ can be decomposed into the following form:

$$\mathbf{B} = \mathbf{I} - \mathbf{D_b}\mathbf{E}, \tag{64}$$

where $\mathbf{I}$ is the $2r \times 2r$ identity matrix, $\mathbf{D_b}$ is a diagonal matrix with diagonal entries

$$[\mathbf{b}_k(B), \mathbf{b}_{k-1}(B), \cdots \mathbf{b}_r(B), \mathbf{b}_r(A), \cdots, \mathbf{b}_{k-1}(A), \mathbf{b}_k(A)],$$

and $\mathbf{E}$ is a matrix that is all ones except for zeros in the last column of the first r rows, and the first column in the last r rows. Formally,

$$\mathbf{E}_{ij} = \begin{cases} 0 & \text{if } j = k \text{ and } i \in \{1, 2, \dots r\} \\ 0 & \text{if } j = 1 \text{ and } i \in \{r+1, r+2, \dots 2r\} \\ 1 & \text{otherwise.} \end{cases}$$

The zero entries reflect the fact that hyperedges of type zero do not affect the affinity scores for class A, and type-k hyperedges do not affect affinity scores for class B.

A primal solution with an objective score of 0 can be obtained by setting $x_j = m_j / (\sum_{i=0}^{k} m_j)$, where m_i is the number of hyperedges of type i in the hypergraph whose affinity scores are equal to the baseline scores $\{\mathbf{b}_i(X)\}$. In order to find a set of dual variables with an objective score of zero, it suffices to find a vector $\mathbf{y}$ that is strictly positive and satisfies $\mathbf{B}^T\mathbf{y}$. Equivalently, given the decomposition of $\mathbf{B}$ in (64), we want $\mathbf{y}$ to satisfy

$$\mathbf{y} = \mathbf{E}^T \mathbf{D}_{\mathbf{b}} \mathbf{y}. \tag{65}$$

Each row and column of $\mathbf{B}$ can be associated with a class X and hyperedge type t, so we doubly index the dual variables as follows

$$\mathbf{y}^T = \begin{bmatrix} y_{B,k} & y_{B,k-1} & \cdots & y_{B,r} & y_{A,r} & \cdots & y_{A,k-1} & y_{A,k} \end{bmatrix}.$$

Rows 2 through $2r-1$ of $\mathbf{E}^T$ have the value 1 in every column, so for this equation to hold we must have

$$y_{X,i} = \sum_{i=r}^{k} \mathbf{b}_i(A) y_{A,i} + \sum_{i=r}^{k} \mathbf{b}_i(B) y_{B,i}. \tag{66}$$

for $i \in \{r, r+1, \cdots, k-1\}$ and $X \in \{A, B\}$. Thus, a necessary condition for satisfying (65) is that entries 2 through $2r-1$ of $\mathbf{y}$ are all equal. With this in mind, let $z > 0$ be a fixed positive value and construct a set of dual variables as follows:

$$\begin{aligned} y_{X,i} &= z \;\; \text{for } i \in \{r, r+1, \cdots, k-1\}, X \in \{A, B\} \\ y_{B,k} &= \frac{z \cdot \sum_{i=r}^{k-1} \mathbf{b}_i(B)}{1 - \mathbf{b}_k(B)} \\ y_{A,k} &= \frac{z \cdot \sum_{i=r}^{k-1} \mathbf{b}_i(A)}{1 - \mathbf{b}_k(A)}. \end{aligned}$$

As long as $z > 0$, we can see that $y_{B,k}$ and $y_{A,k}$ will also be strictly positive. We can also set z so that the entries of $\mathbf{y}$ sum to one. As long as we can show $\mathbf{B}^T\mathbf{y} = 0$, this means we have a set of dual variables with an objective score of zero, confirming that majority homophily cannot hold for both classes simultaneously.

Variables $y_{B,k}$ and $y_{A,k}$ are explicitly chosen so that the first and last entries in equation (65) hold when all other variables are equal to a positive constant z. To check that $\mathbf{B}^T\mathbf{y} = 0$, we just need to confirm that (66) holds. Recall that the baseline scores can be written in terms of hyperedge counts for some hypergraph G, i.e., for $i \in [k]$

$$\begin{aligned} \mathbf{b}_i(A) &= \frac{m_i}{D_A} \\ \mathbf{b}_i(B) &= \frac{m_{k-i}}{D_B} \end{aligned}$$

where $D_A = \sum_{i=1}^{k} m_i$ and $D_B = \sum_{i=1}^{k} m_{k-i} = \sum_{i=0}^{k-1} m_i$. Notice that $D_A - m_k = D_B - m_0 = \sum_{i=1}^{k-1} m_i$.

Substituting our choice of variables into the right hand side of (66), we confirm that the equation holds:

$$
\begin{aligned}
&\sum_{i=r}^{k} \mathbf{b}_i(A) y_{A,i} + \sum_{i=r}^{k} \mathbf{b}_i(B) y_{B,i} \\
&= z \cdot \sum_{i=r}^{k-1} \mathbf{b}_i(A) + z \cdot \sum_{i=r}^{k-1} \mathbf{b}_i(B) + \mathbf{b}_k(A) y_{A,k} + \mathbf{b}_k(B) y_{B,k} \\
&= z \cdot \sum_{i=r}^{k-1} \mathbf{b}_i(A) + z \cdot \sum_{i=r}^{k-1} \mathbf{b}_i(B) + \mathbf{b}_k(A) \frac{z \cdot \sum_{i=r}^{k-1} \mathbf{b}_i(A)}{1 - \mathbf{b}_k(A)} + \mathbf{b}_k(B) \frac{z \cdot \sum_{i=r}^{k-1} \mathbf{b}_i(B)}{1 - \mathbf{b}_k(B)} \\
&= z \cdot \sum_{i=r}^{k-1} \mathbf{b}_i(A) \left(1 + \frac{\mathbf{b}_k(A)}{1 - \mathbf{b}_k(A)}\right) + z \cdot \sum_{i=r}^{k-1} \mathbf{b}_i(B) \left(1 + \frac{\mathbf{b}_k(B)}{1 - \mathbf{b}_k(B)}\right) \\
&= z \cdot \quad \sum_{i=r}^{k-1} \frac{m_i}{D_A} \left(\frac{1}{1 - \mathbf{b}_k(A)}\right) + \sum_{i=r}^{k-1} \frac{m_{k-i}}{D_B} \left(\frac{1}{1 - \mathbf{b}_k(B)}\right)\Bigg) \\
&= z \cdot \quad \sum_{i=r}^{k-1} \frac{m_i}{D_A} \left(\frac{D_A}{D_A - m_k}\right) + \sum_{i=r}^{k-1} \frac{m_{k-i}}{D_B} \left(\frac{D_B}{D_B - m_0}\right)\Bigg) \\
&= z \cdot \quad \sum_{i=r}^{k-1} \frac{m_i + m_{k-i}}{\sum_{i=1}^{k-1} m_i}\Bigg) \\
&= z \cdot 1 = z.
\end{aligned}
$$

□

An analogous impossibility result also holds for even k when we use alternative affinity scores.

Theorem 13. *If k is even, it is impossible for both classes to exhibit monotonic homophily if additionally $\mathbf{a}_\ell(X) > \boldsymbol{b}_\ell(X)$ for one class $X \in \{A, B\}$ when $\ell = k/2$.*

Proof. The proof is nearly identical to the proof of Theorem 12. For even k, let $r = \frac{k}{2} + 1$. We use the same linear program encoding the maximum possible amount of majority homophily, with an additional constraint for class A:

$$x_\ell - g_\ell(A) \cdot \sum_{i=1}^{k} x_i \geq \gamma.$$

The LP and its dual can again be written in the form (60) and (61). The matrix $\mathbf{B}$ is given by

$$\mathbf{B} = \mathbf{I} - \mathbf{D_b}\mathbf{E},$$

where $\mathbf{I}$ is the $(k+1) \times (k+1)$ identity matrix, $\mathbf{D_b}$ is a diagonal matrix with diagonal entries

$$[\mathbf{b}_k(B), \mathbf{b}_{k-1}(B), \cdots, \mathbf{b}_r(B), \mathbf{b}_\ell(A), \mathbf{b}_r(A), \cdots, \mathbf{b}_{k-1}(A), \mathbf{b}_k(A)],$$

and $\mathbf{E}$ is a matrix that is all ones except for zeros in the last column of the first $k/2$ rows, and zeros in the first column in the last $k/2 + 1$ rows. The dual variables of the linear program can be indexed as follows

$$\mathbf{y}^T = \begin{bmatrix} y_{B,k} & y_{B,k-1} & \cdots & y_{B,r} & y_{A,\ell} & y_{A,r} & \cdots & y_{A,k-1} & y_{A,k} \end{bmatrix}.$$

The proof again follows as long as we can find a nonnegative vector $\mathbf{y}$ satisfying $\mathbf{B}^T\mathbf{y} = 0$, or equivalently $\mathbf{y} = \mathbf{E}^T\mathbf{D}_\mathbf{b}\mathbf{y}$. In order to accomplish this, all but the first and last dual variable can be set equal to some positive value z, and then we can solve for $y_{A,k}$ and $y_{B,k}$:

$$y_{B,k} = \frac{z \cdot \sum_{i=r}^{k-1} \mathbf{b}_i(B)}{1 - \mathbf{b}_k(B)}$$
$$y_{A,k} = \frac{z \cdot \sum_{i=\ell}^{k-1} \mathbf{b}_i(A)}{1 - \mathbf{b}_k(A)}.$$

The only difference from the dual variables in Theorem 12 is that the summation in the numerator of $y_{A,k}$ starts from ℓ rather than from r. The rest of the result follows by showing algebraically that $\mathbf{B}^T\mathbf{y} = 0$. □

4 Dataset Information and Additional Experimental Results

In this section we provide details regarding the datasets used in the main text and additional experimental results. Further information about each dataset is available from the original source. For all experiments in the main text and the supplement, we used asymptotic baseline scores when forming the ratio plots (see the Materials and Methods section). Code and data sufficient to reproduce all experimental results in the main text and in the supplement is available on Zenodo (`https://doi.org/10.5281/zenodo.7086798`) as well as on GitHub (`https://github.com/nveldt/HypergraphHomophily`).

In order to provide an additional comparison against graph-based approaches, we compute and report graph homophily indices obtained by projecting each hypergraph into a graph based on co-participation in group interactions. These graph homophily indices do provide some information regarding the level of homophily exhibited by each class of nodes in each dataset, but they do not allow us to capture any notion of majority or monotonic homophily. These graph scores also often hide the way in which group size affects the level of homophily that is exhibited by a class.

Co-authorship and gender The co-authorship dataset we considered is the DBLP Records and Entries for Key Computer Science Conferences [33], originally used in a study on women in computer science research [34] and available online at `https://data.mendeley.com/datasets/3p9w84t5mr/1`. The data constitutes 16 years of publications at top computer science conferences, from 2000 to 2015, listed on the DBLP bibliography database. We consider only authors in the dataset whose gender is known with high confidence, and discard all papers including an author of unknown gender. The resulting hypergraph has 105256 nodes (82620 male, 22636 female), with a maximum hyperedge size of 21. The number of hyperedges between size 2 and 4 is 74134.

We can project the hypergraph into a graph by including an edge between author i and j if they ever publish a paper together. If we perform this projection using all group interactions (i.e., all papers), the resulting graph has a homophily index of 0.270 for women and a homophily index of 0.821 for men. If we project only group interactions with 2-4 authors, the scores are similar: 0.261 and 0.828. In both cases, the scores are higher than the relative class proportions of 0.215 and 0.785.

Congress bills and political parties The congress bills dataset is made up of legislative bills co-sponsored by US politicians in the Senate and House of Representatives. The original data was collected by James Fowler [24,26]. For our experiments we consider a derivation of the dataset presented as timestamped hyperedges [25], available online at `https://www.cs.cornell.edu/~arb/data/congress-bills`. The hypergraph has 1718 nodes (810 Republican and 908 Democrat) and 83105 hyperedges ranging from size 2 to 25.

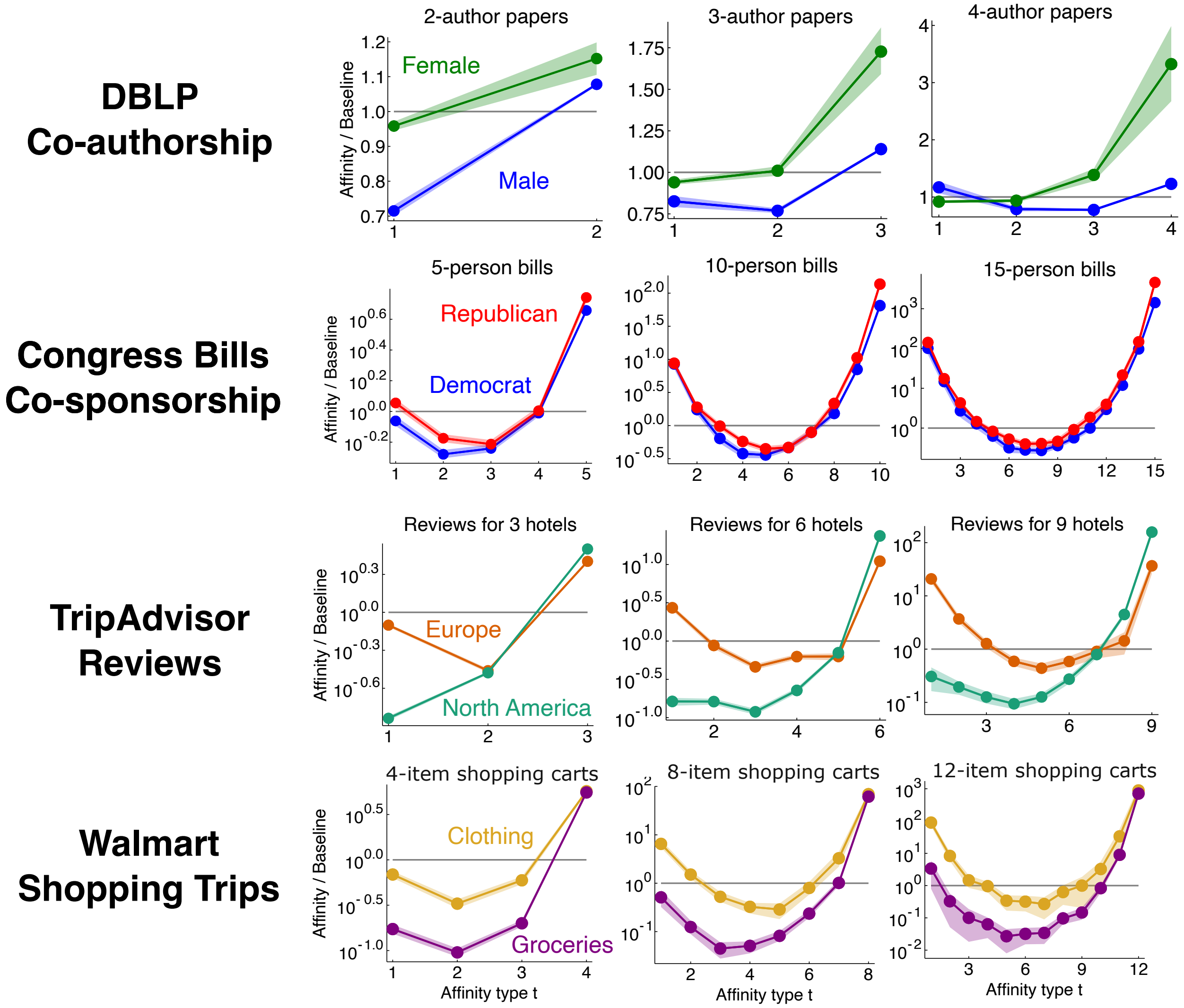

Figure S1: **Results from running a bootstrapping procedure to check the robustness of empirical results.** Ratio scores are shown for the DBLP co-authorship hypergraph (top row), the congress bills hypergraph (second row), the TripAdvisor hypergraph (third row), and the walmart hypergraph (last row). These results indicate that affinity and ratio scores in each case are robust to perturbations in the data. For each hypergraph and a range of values of k, the procedure samples from the original set of size-k hyperedges with replacement, and computes affinity scores and ratio scores each time. Solid lines in the plots indicate the true affinity scores computed on the entire dataset, which are nearly identical to the mean affinity score obtained from 100 runs of this procedure. Lighter colored regions show two standard errors above and below the mean. In many plots, the error region is too small to be perceptible.

If we project all group interactions to a graph, the graph homophily indices for Republicans and Democrats are 0.499 and 0.591, respectively, and their class proportions are 0.471 and 0.529. In other words, graph homophily indices are only slightly above baseline. If we only consider group interactions of size 20 or smaller (the largest group size we considered for our hypergraph affinity scores in the main text), the homophily indices increase only slightly to 0.516 and 0.606. These scores are obtained if we use an unweighted graph projection, where nodes i and j have a unit weight edge if they ever co-participate in a hyperedge. We can also perform a weighted projection where the weight of an edge between nodes i and j equals the *number* of hyperedges they both participate in. If we perform this weighted projection for all groups of size up to 20, graph homophily indices for Republicans and Democrats are 0.586 and 0.724 respectively. These higher

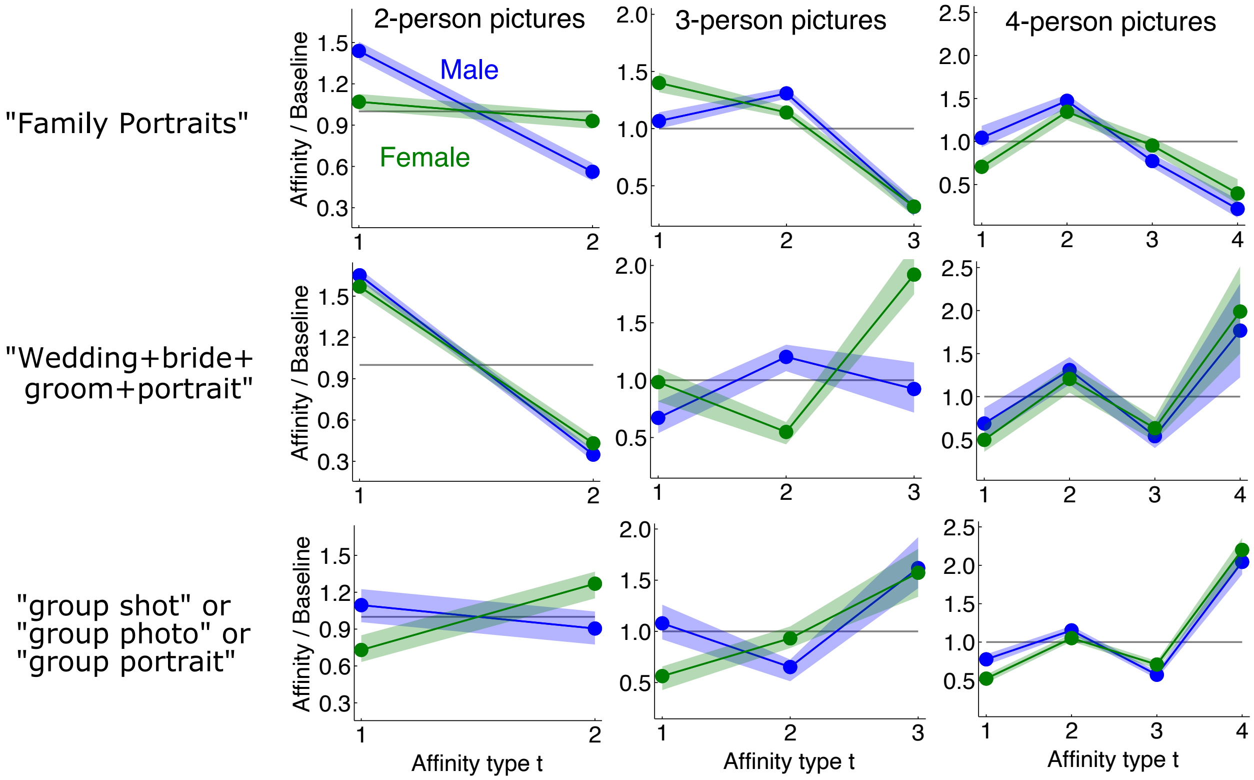

Figure S2: **Ratio scores obtained from a bootstrapping procedure on the group pictures dataset, indicating the robustness of our results for each group picture type.** The solid lines indicate true affinity scores, which are very close to the mean affinity score from taking 100 different samples from the data. Lighter colored regions show one standard error above and below the mean from this bootstrapping procedure. On the subsampled datasets, we continue to see all the same major trends and differences between pictures types as we do when using all of the data.

scores indicate that it is common to see *repeated* collaborations between the same two individuals from the same political party, which is also a valid notion of homophily. This is arguably a more accurate measure of the high levels of homophily present in the dataset, though this still does not capture the extreme notions of majority and monotonic group homophily that we reveal using our hypergraph measures.

TripAdvisor hotels and locations The TripAdvisor hypergraph is derived from a review dataset originally used for research on opinion mining from online reviews [27], available online at `https://www.cs.virginia.edu/~hw5x/dataset.html`. We associate each hotel in the dataset from North America or Europe as a labeled node in a hypergraph. We discard reviews from the original data that are cross-listed reviews from other travel sites, and reviews where the reviewer name is simply "A TripAdvisor Member". For each remaining unique reviewer, we construct a hyperedge joining all hotels they reviewed. The resulting hypergraph has 8956 nodes and 130570 hyperedges of size 2 to 87. The main text shows MaHI and MoHI scores for group sizes up to $k = 13$. If we project groups of size 2 to 13 to an unweighted graph, the graph homophily indices for North American and Europe are 0.829 and 0.519, respectively. If we use a weighted graph projection, graph homophily indices increase to 0.893 and 0.613. In both cases, graph homophily indices are well above baseline class proportions of 0.502 and 0.498.

Walmart trips dataset The Walmart products datasets is available online at `https://www.kaggle.com/c/walmart-recruiting-trip-type-classification`. This dataset was first made avail-

Table S1: **Graph homophily indices obtained by projecting three different types of group picture datasets to graphs**. We report indices obtained from projecting groups of size 2, 3, and 4, separately. We also provide graph homophily indices obtained when projecting multiple groups sizes at once to a graph.

	Family Pictures			Wedding Pictures			General Group Pictures		
k	women	men	α_W / α_M	women	men	α_W / α_M	women	men	α_W / α_M
2	0.465	0.281	0.57 / 0.43	0.215	0.174	0.51 / 0.49	0.635	0.452	0.6 / 0.4
3	0.365	0.406	0.48 / 0.52	0.617	0.531	0.55 / 0.45	0.626	0.567	0.54 / 0.46
4	0.456	0.405	0.52 / 0.48	0.557	0.519	0.52 / 0.48	0.583	0.543	0.52 / 0.48
2-4	0.426	0.395	0.52 / 0.48	0.452	0.394	0.52 / 0.48	0.588	0.543	0.53 / 0.47
2-10	0.433	0.411	0.51 / 0.49	0.571	0.546	0.52 / 0.48	0.599	0.583	0.51 / 0.49

able as part of a Kaggle competition. Amburg et al. [28] derived a large hypergraph of co-purchased products based on the data, and identified department labels for each product. This derived hypergraph is available at https://www.cs.cornell.edu/~arb/data/walmart-trips/. In our work, we restrict to the subset of grocery and clothes products, resulting in a hypergraph with 48480 nodes (26178 grocery products, and 22302 clothes products) and 47034 hyperedges of size 2 to 25.

The main text shows MaHI and MoHI scores for group sizes up to $k = 14$. If we project groups of size 2 to 14 to an unweighted graph, the graph homophily indices for Groceries and Clothing are 0.942 and 0.618, respectively. If we use a weighted graph projection, these indices increase only slightly to 0.948 and 0.621. Baseline scores (i.e., class proportions) are 0.540 and 0.460 for Groceries and Clothing, respectively.

Group pictures and gender The three different group picture datasets [29] were downloaded from http://chenlab.ece.cornell.edu/people/Andy/ImagesOfGroups.html. Images in the original dataset were obtained via three different Flickr search queries, producing sets of family pictures, wedding pictures, and general group pictures. We parse and store data for all group pictures with up to $k = 10$ people. The number of pictures with between two and four people for each type of group picture is 1051, 662, and 963 for family, wedding, and general group pictures respectively. We used even baseline scores for the experiments, which assumes an equal number of men and women. Although we do not have unique identifiers for people in the pictures, we checked that the data is indeed roughly gender balanced. Treating each person in each picture as unique, 51-52% of the subjects are female in each of the three group picture datasets.

In Figure 6 of the main text, the graph homophily indices we report are based on projecting all group pictures with up to 10 people to a graph based on co-appearance. For example, we reported graph homophily indices of 0.57 and 0.55 (for women and men, respectively) for wedding pictures. If we only project hyperedges of size 2 to 4 for wedding pictures, then the affinity scores are instead 0.45 for women and 0.40 for men, which are just below baseline values. Whether we project using all groups pictures or only small group pictures ($k = 2, 3, 4$), reducing group interactions to pairwise co-appearances overlooks meaningful information about the way in which homophily depends on the group size. One way to partially remedy this issue is to separately project different group sizes to different graphs, and then compute a graph homophily index for each separate graph. In Table S1, we report the graph homophily indices obtained by projecting size 2, 3, and 4 group pictures to a graph, as well as scores obtained from projecting all groups of size 2-4, and all groups of size 2-10. Columns α_W and α_M represent the class proportion (i.e., baseline scores) for women and men, respectively. This approach of separately projecting different group sizes is already a departure from standard graph-based techniques for measuring homophily, which often project all

group sizes at once. These separate graph projections do provide one way to observe the way homophily depends on group size. In wedding pictures, for example, these separated scores accurately capture the fact that homophily is not present for groups of size $k = 2$, but starts to become more present for groups of size $k = 3$ and $k = 4$. However, separately projecting different group sizes still does not capture the fact that ratio scores meaningfully differ within each group size k depending on affinity type $t \leq k$. For example, our hypergraph scores capture the fact that in wedding group pictures with $k = 4$ people, type-3 affinity scores are below baseline while groups that are perfectly gender homogeneous or perfectly gender balanced are above baseline. Similarly, in family pictures with 4 people, our hypergraph measures capture the fact that type-2 affinities are above baseline, which intuitively reflects a high proportion of 4-person pictures of a husband, wife, one boy, and one girl. Graph homophily indices do not capture this observation.

4.1 Checking Robustness of Affinity Scores

By design, the affinity scores and the impossibility results we have considered apply to a specific hypergraph with a fixed set of hyperedges. In the main text, we therefore used all available hyperedge information when plotting results for each dataset we considered. Building a hypergraph from real data can be a noisy and imperfect process, and affinity scores will therefore vary depending on the quality of data and availability of group information in each context. We use a simple bootstrapping procedure to show that the basic patterns in affinity, ratio, and normalized bias scores for all of the hypergraphs we consider are stable to perturbations in the data. Given a hypergraph H with m_k hyperedges of size k, we sample m_k of these hyperedges with replacement and compute typed affinity, ratio, and normalized bias scores from the set of sampled hyperedges in each case. We repeat the process 100 times for each dataset, and then compute the mean and standard error for the resulting ratio scores. Figures S1 and S2 display bootstrapping results for ratio scores. The same observations about robustness apply to affinity scores and normalized bias scores as well. Results on paper co-authorship, congress bill co-sponsorship, TripAdvisor reviews, and Walmart shopping trips are particularly robust to perturbations in the data. Average scores when bootstrapping are nearly identical to scores obtain when using the entire hypergraph, and there is a very small standard error (Fig. S1). Scores for group pictures vary slightly more depending on the sample, but still preserve the same shape and pattern. We observe the same basic differences among pictures with regard to group type (wedding, family, or general group picture) and size (Fig. S2).

4.2 Experimental Results on Contact Data

In addition to the empirical results shown in the main text, we compute affinity scores with respect to gender for group gatherings among primary and high schools students [25, 35, 36]. Each hypergraph consists of groups of students (in primary school [36] and high school [35] respectively) that gathered together in close proximity at some point during the day, as measured by wearable sensors. In high school student interactions of size two through four (Fig. S3), students have high tendencies for gathering in groups where all members are of the same gender. All other affinity scores are below baseline. Results for primary school interactions are nearly the same (Fig. S4), except in the case of groups of size two, where groups with two male students are just slightly below baseline. We applied bootstrapping to both datasets (see Section 4.1 for details), to confirm that our results are robust to perturbations in the data. For both hypergraphs we observe a small standard error around the mean affinity scores obtained across different subsamples of the hyperedges.

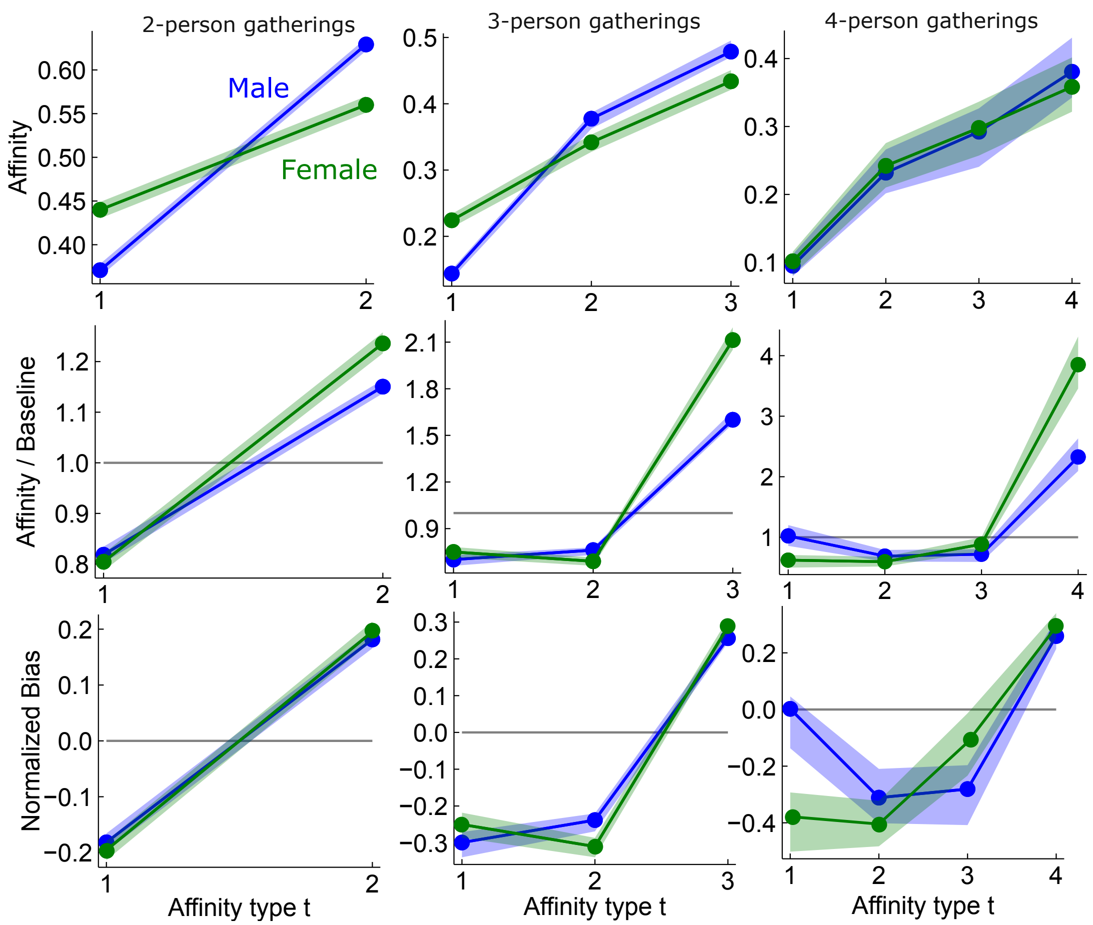

Figure S3: **Affinity, ratio, and normalized bias scores with respect to gender for a contact hypergraph at a high school**. Nodes in the hypergraph are students, and hyperedges indicate sets of students who were gathered in close proximity at some point, as measured by wearable sensors. Solid lines indicate affinity scores for the entire hypergraph, and lighter colored regions show one standard error around the mean affinity score obtained from a bootstrapping procedure on the hyperedges, indicating our results are robust to perturbations in the data. Both genders exhibit strong simple homophily, meaning a high tendency for gathering in groups where everyone is of the same gender. All other scores are below baseline. For gatherings of size three and four, ratio scores almost increase monotonically, though perfect monotonic increase is impossible, as shown by our theoretical results. However, monotonic increase in raw affinity scores is possible, as seen in plots from the first row. This results from having a roughly balanced number of gatherings of each type.

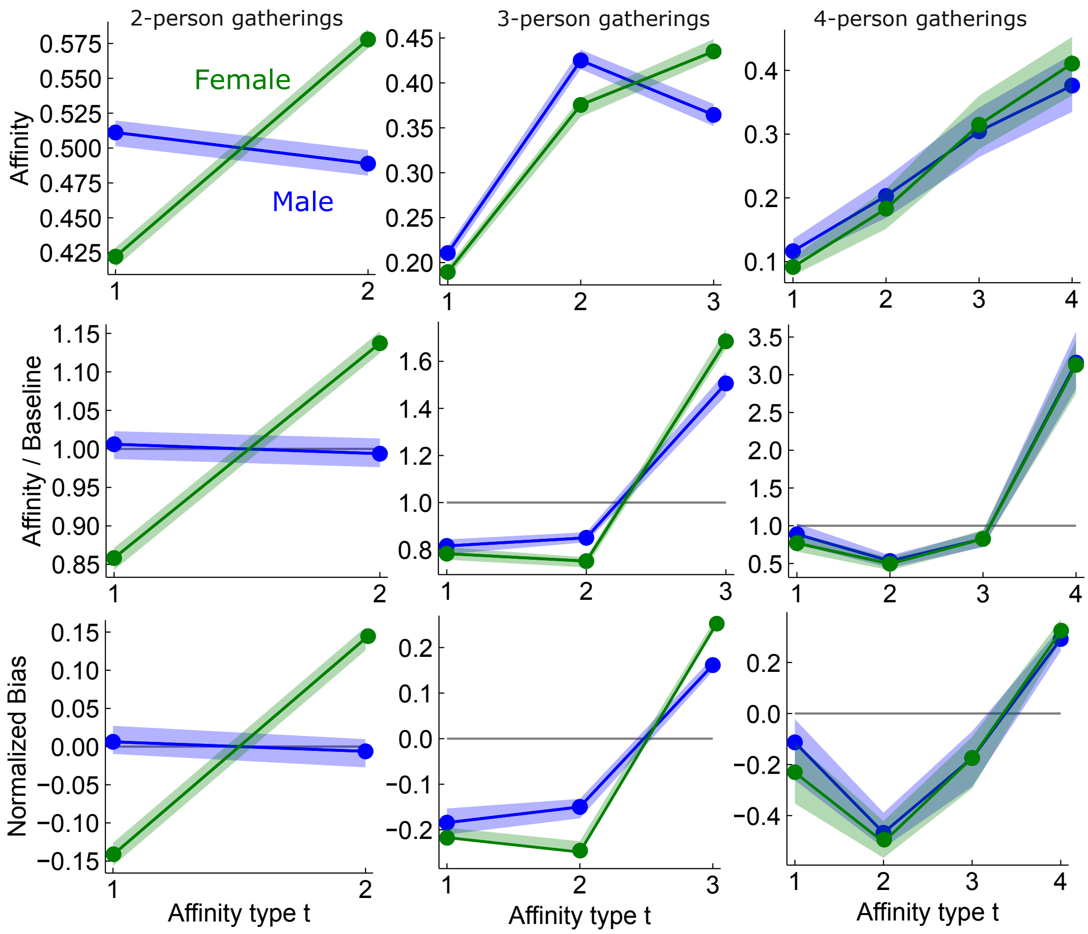

Figure S4: **Affinity, ratio, and normalized bias scores with respect to gender for a contact hypergraph at a primary school**. Hyperedges indicate a set of students (nodes) who were gathered in close proximity at some point, as measured by wearable sensors. Solid lines again indicate affinity scores for the entire dataset, and lighter colored regions show one standard error around the mean affinity score from a bootstrapping. For groups of size three and four, both genders exhibit strong simple homophily, indicating a high tendency towards groups where everyone is of the same gender. For groups of size two, male students have affinity scores that are almost exactly equal to baseline.

REFERENCES AND NOTES

1. P. Lazarsfeld, R. K. Merton, Friendship as a social process: A substantive and methodological analysis, in *Freedom and Control in Modern Society* (Van Nostrand, 1954), p. 18–66.

2. J. Moody, Race, school integration, and friendship segregation in america. *Am. J. Sociol.* **107**, 679–716 (2001).

3. W. Shrum, N. H. Cheek, S. MacD. Hunter, Friendship in school: Gender and racial homophily. *Sociol. Educ.* **61**, 227–239 (1988).

4. P. V. Marsden, Homogeneity in confiding relations. *Soc. Netw.* **10**, 57–76 (1988).

5. C. P. Loomis, Political and occupational cleavages in a Hanoverian Village, Germany: A sociometric study. *Sociometry* **9**, 316–333 (1946).

6. J. C. Almack, The influence of intelligence on the selection of associates. *Sch. Soc.* **16**, 529–530 (1922).

7. H. M. Richardson, Community of values as a factor in friendships of college and adult women. *J. Soc. Psychol.* **11**, 303–312 (1940).

8. M. Kalmijn, Intermarriage and homogamy: Causes, patterns, trends. *Annu. Rev. Sociol.* **24**, 395–421 (1998).

9. L. M. Verbrugge, A research note on adult friendship contact: A dyadic perspective. *Soc. Forces* **62**, 78–83 (1983).

10. B. H. Mayhew, J. Miller McPherson, T. Rotolo, L. Smith-Lovin, Sex and race homogeneity in naturally occurring groups. *Soc. Forces* **74**, 15–52 (1995).

11. J. M. Cohen, Sources of peer group homogeneity. *Sociol. Educ.* **50**, 227–241 (1977).

12. M. McPherson, L. Smith-Lovin, J. M. Cook, Birds of a feather: Homophily in social networks. *Annu. Rev. Sociol.* **27**, 415–444 (2001).

13. J. Miller McPherson, T. Rotolo, Testing a dynamic model of social composition: Diversity and change in voluntary groups. *Am. Sociol. Rev.* **61**, 179–202 (1996).

14. J. Miller McPherson, L. Smith-Lovin, Homophily in voluntary organizations: Status distance and the composition of face-to-face groups. *Am. Sociol. Rev.* **52**, 370–379 (1987).

15. R. L. Breiger, The duality of persons and groups. *Soc. Forces* **53**, 181–190 (1974).

16. K. M. Altenburger, J. Ugander, Monophily in social networks introduces similarity among friends-of-friends. *Nat. Hum. Behav.* **2**, 284–290 (2018).

17. S. Currarini, M. O. Jackson, P. Pin, An economic model of friendship: Homophily, minorities, and segregation. *Econometrica* **77**, 1003–1045 (2009).

18. J. Coleman, Relational analysis: The study of social organizations with survey methods. *Hum. Organ.* **17**, 28–36 (1958).

19. M. E. J. Newman, Mixing patterns in networks. *Phys. Rev. E* **67**, 026126 (2003).

20. M. Gentzkow, J. M. Shapiro, M. Taddy, Measuring group differences in high-dimensional choices: Method and application to congressional speech. *Econometrica* **87**, 1307–1340 (2019).

21. L. A. Adamic, N. Glance, The political blogosphere and the 2004 U.S. election: Divided they blog, in *Proceedings of the 3rd International Workshop on Link Discovery*, LinkKDD ’05, Association for Computing Machinery, (New York, NY, USA, 2005), p. 36–43..

22. K. T. Poole, H. Rosenthal, A spatial model for legislative roll call analysis. *Am. J. Polit. Sci.*, **29**, 357–384 (1985).

23. B. R. Berelson, P. F. Lazarsfeld, W. N. McPhee, *Voting: A Study of Opinion Formation in a Presidential Campaign* (University of Chicago Press, 1954).

24. J. H. Fowler, Legislative cosponsorship networks in the US house and senate. *Soc. Netw.* **28**, 454–465 (2006).

25. A. R. Benson, R. Abebe, M. T. Schaub, A. Jadbabaie, J. Kleinberg, Simplicial closure and higher-order link prediction. *Proc. Natl. Acad. Sci. U.S.A.* **115**, E11221–E11230 (2018).

26. J. H. Fowler, Connecting the congress: A study of cosponsorship networks. *Polit. Anal.* **14**, 456–487, 2006.

27. I. Amburg, N. Veldt, A. R. Benson, Clustering in graphs and hypergraphs with categorical edge labels, in *Proceedings of the Web Conference* (2020), p. 706–717.

28. A. C. Gallagher, T. Chen. Understanding images of groups of people, in *Proceedings of the 2009 IEEE Conference on Computer Vision and Pattern Recognition* (IEEE, 2009), p. 256–263.

29. H. Wang, Y. Lu, C. Zhai, Latent aspect rating analysis without aspect keyword supervision, in *Proceedings of the 17th ACM SIGKDD international conference on Knowledge discovery and data mining* (ACM, 2011), pages 618–626.

30. D. Ghoshdastidar, A. Dukkipati, Consistency of spectral partitioning of uniform hypergraphs under planted partition model, in *Advances in Neural Information Processing Systems* (2014), p. 397–405.

31. R. Van Der Hofstad, *Random Graphs and Complex Networks* (Cambridge Univ. Press, 2016), vol. 1.

32. R. J. Serfling. Some elementary results on Poisson approximation in a sequence of Bernoulli trials. *SIAM Rev.* **20**, 567–579 (1978).

33. S. Agarwal, A. Sureka, N. Mittal, R. Katyal, D. Correa, DBLP records and entries for key computer science conferences. Mendeley Data, V1, 2016.

34. S. Agarwal, N. Mittal, R. Katyal, A. Sureka, D. Correa, Women in computer science research *SIGCAS Comput. Soc.* **46**, 7–19 (2016).

35. R. Mastrandrea, J. Fournet, A. Barrat, Contact patterns in a high school: A comparison between data collected using wearable sensors, contact diaries and friendship surveys. *PLOS ONE* **10**, e0136497 (2015).

36. J. Stehlé, N. Voirin, A. Barrat, C. Cattuto, L. Isella, J.-F. Pinton, M. Quaggiotto, W. Van den Broeck, C. Régis, B. Lina, P. Vanhems, High-resolution measurements of face-to-face contact patterns in a primary school. *PLOS ONE* **6**, e23176 (2011).
